# Integrated stress signaling pathways promote adaptive reprogramming for chondrocyte survival in Metaphyseal Chondrodysplasia Type Schmid

**DOI:** 10.1038/s41413-026-00587-5

**Published:** 2026-10-16

**Authors:** Qing Yao, Wilson Cheuk Wing Chan, Yuliana Sandraling Lie, Cheng Wang, Vivian Chor Wing Ng, Zhijia Tan, Steven Lim Cho Pei, Ning Chen, Michael Kai Tsun To, Kathryn Song Eng Cheah, Danny Chan

**Affiliations:** 1 School of Biomedical Sciences, Li Ka Shing Faculty of Medicine, The University of Hong Kong, Pokfulam, Hong Kong SAR, China; 2 School of Biomedical Engineering, Li Ka Shing Faculty of Medicine, The University of Hong Kong, Pokfulam, Hong Kong SAR, China; 3 Clinical Research Center for Rare Diseases, The University of Hong Kong-Shenzhen Hospital, Shenzhen, China; 4 Department of Orthopaedics and Traumatology, The University of Hong Kong-Shenzhen Hospital, Shenzhen, China

**Keywords:** Bone, Homeostasis

## Abstract

Collagen X, encoded by the *Col10a1* gene, is expressed specifically by hypertrophic chondrocytes (HCs) in the cartilage. Mutations of the *Col10a1* gene in humans, which increase the endoplasmic reticulum (ER) stress in HCs, lead to metaphyseal chondrodysplasia type Schmid (MCDS). However, the mechanism of how HCs respond to ER stress to affect bone development and growth is not entirely understood. In this study, we generated a new mouse model harboring a 13 bp deletion (13del) in the endogenous *Col10a1* gene. These 13del-MCDS mice display all the known clinical characteristics of human MCDS, as well as the molecular and cellular changes within the growth plate cartilage. For the first time, we found that triggering ER stress leads to activation of hypoxia and HIFs pathways in vivo, promoting cellular survival in HCs of the 13del-MCDS mice. To test whether the activated hypoxia and HIFs pathways are triggered by ER stress or extrinsic factors, we generated WT/GFP-MCDS chimera mice. Our findings showed that the activation of hypoxia and the hypoxic pathway is an intrinsic response to ER stress, which may partly occur through activation of ATF4 in HCs of mice. In parallel, p53 pathway changes and autophagy-associated protein-quality-control responses were observed in spatial association with altered chondrocyte differentiation and survival. These findings reveal coordinated engagement of multiple stress-response pathways during the adaptive response of hypertrophic chondrocytes to chronic ER stress.

## Introduction

Metaphyseal Chondrodysplasia Type Schmid (MCDS, OMIM#156500) is a rare autosomal dominant skeletal disorder characterized by short stature with short limbs*, coxa vara, genu varum*, and waddling gait.^1–3^ MCDS patients generally appear normal at birth, and the onset of clinical symptoms usually occurs when they begin to walk. MCDS is associated with mutations in the *COL10A1* gene, with an estimated prevalence of between 3 and 6 cases per million.^1^ There is no cure for MCDS; therefore, treatment focuses on managing symptoms, correcting deformities, and providing supportive care to improve quality of life.^4^ Within the cartilage growth plate of growing bones, COL10A1 is expressed by hypertrophic chondrocytes (HCs) during the process of endochondral ossification, a process essential for the linear growth of long bones.^5^

The *COL10A1* gene encodes the α1(X)-chain of Collagen X, which folds into a homotrimer protein localized to the mineralizing hypertrophic cartilage of the growth plate.^5^ Numerous mutations in *COL10A1* have been identified as associated with MCDS,^6^ with the (*c.1857_1869del13*) mutation as the first one identified.^2^ Collagen X is a short-chain collagen that consists of a short triple helical domain flanked by non-collagenous NC1 and NC2 domains at the C- and N-terminus, respectively.^7^ With the exception of two missense mutations in the signal peptide cleavage site of the NC2 domain,^8,9^ the majority of reported mutations were identified within the NC1 domain of the gene,^7,10,11^ consisting of missense, nonsense, and deletion mutations that result in an abnormal NC1 domain. Given that the NC1 domain functions in the initiation of trimer formation, these mutations impair trimerization^12,13^ and secretion from the endoplasmic reticulum (ER), activating ER stress as a pathological outcome from in vitro and in vivo studies.^12,14,15^

Studies in mouse models have implicated ER-stress signaling in the pathogenesis of MCDS, confirming activation of ER-stress as the primary pathological outcome causing the disease. These studies included transgenic mouse models with overexpression of mouse *Col10a1* in HCs containing human-equivalent MCDS NC1 mutations. These transgenic mice exhibited characteristics of MCDS with the following mutations: *p.P620fsX621* (FCel-Tg)^15^ and 13 bp frameshift (13del-Tg) (*c.1857_1869del13*) mutation.^14^ In these models, mutant Collagen X proteins were produced by HCs, leading to misfolding and retention of unfolded Collagen X within the ER, which activated unfolded protein response (UPR) pathways as part of the ER stress signaling (ERSS), showing activation of all the UPR sensors, IRE1, PERK, and ATF6.^14^ These sensors are activated as an immediate response to alleviate or cope with stress as a survival strategy.^16–18^ Activation of ER stress can also result in cell death under chronic conditions.^17,18^ Strong evidence supporting ER stress activation as a primary cause of MCDS comes from a study of a transgenic mouse line using the *Col10a1* promoter to drive expression of an ER stress-inducing protein (the cog mutant of thyroglobulin) in HCs, resulting in an MCDS-like phenotype.^19^

The cartilage growth plate consists of three progressive zones: the reserve, proliferative, and hypertrophic zones.^5^ The reserve zone is a source of chondrocytes entering the proliferative zone that subsequently undergo hypertrophy, and the mineralizing hypertrophic cartilage is converted to bone as part of the process of endochondral ossification.^5^ In all MCDS mouse models, a major and consistent pathology of the cartilage growth plate is an expansion of the hypertrophic zone, caused by a delay in the conversion to bone due to survival and “reprogramming” or reversion of the HCs to a pre-hypertrophic chondrocyte state.^14,15^ However, the mechanism by which HCs respond to ER stress affecting bone development and growth remains unclear. A recent study has shown that the integrated stress response (ISR) is part of the mechanism, involving activation of the PERK sensor and the ATF4 axis, resulting in an up-regulation of *Sox9* and promoting the reprogramming process.^20^

Most studies used transgenic mice for the overexpression of MCDS *Col10a1* alleles. In these transgenic models, mutant allele expression was predominantly in HCs but was not fully restricted to this cell population, with ectopic expression observed at lower levels in other cells, such as osteocytes.^21^ So far, there are two MCDS mouse models with gene-targeted MCDS alleles carrying human pN617K^22^ and p.Y632X^23^ mutations in the NC1 domain introduced into the mouse *Col10a1* locus. While these mouse models exhibited growth plate abnormalities, they did not show all the known features of human MCDS.

Given the findings on the role of abnormal protein folding and the activation of UPR as a mechanism, resolving protein folding and/or alleviating ER stress were tested as potential therapeutic options for treating MCDS. Indeed, the use of carbamazepine (CBZ), a drug that stimulates intracellular proteolysis and alleviates ER stress, did effectively reduce the disease severity in the p.Y632X model caused by a premature stop codon in the *Col10a1* gene.^23^ Further, treatment of the 13del-Tg mice targeting the ISR early at the PERK sensor pathway with a small-molecule, integrated stress response inhibitor (ISRIB), resulted in a significant improvement of the skeletal abnormalities.^20^ These findings are very encouraging, prompting the need for more thorough investigations into the cellular survival mechanism in MCDS mice.

Here, we report the generation of a novel gene-targeted mouse model (13del-MCDS) harboring the same 13 bp deletion in the NC1 domain (c.1857_1869del13) that was used to generate the 13del-Tg mouse.^14^ The 13del-MCDS mice exhibited all known characteristics of human MCDS patients, representing a “true model” of MCDS. We also showed activation of the p53, Hif1α, and autophagy pathways in the HCs of 13del-MCDS mice. These findings provide insights into the integrated relationship between survival signals of cellular stress pathways and re-differentiation of HCs under ER stress signaling (ERSS) in the pathogenesis of MCDS, and potential additional therapeutic entry points.

## Results

### The 13del-MCDS mouse exhibits all reported characteristics of human MCDS

**Fig. 1:**
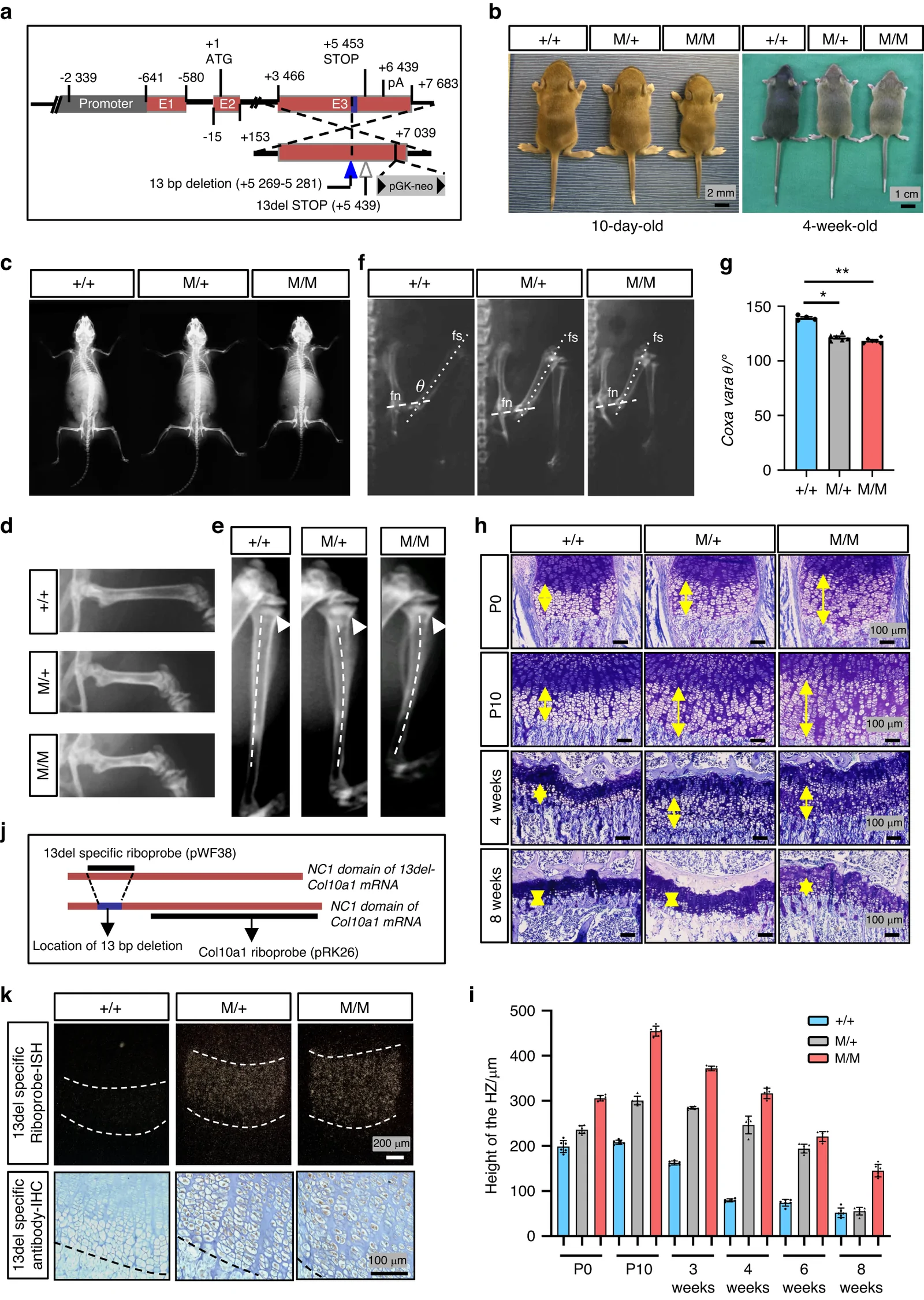
Skeletal and growth plate phenotypes of the 13del-MCDS mouse. **a** Targeting construct for the generation of 13del-MCDS mouse (E=exons). **b** Representative gross appearances of wild type (+/+), heterozygous (M/+) and homozygous (M/M) 13del-MCDS mice at 10 days and 4 weeks of age (*n* = 6). **c** Radiographs of 8-week-old 13del-MCDS mice showing the overall skeletal features. **d** Shortening of femurs and **e** bowing of the tibias and flaring of the metaphyses (white arrows) in M/+ and M/M mice. **f** *Coxa vara* depicted by the reduced angles (θ) (g) (θ) between the femur neck (fn) and shaft (fs) in M/+ and M/M (*n* = 6). **h** Histological sections of the tibia of P0, P10, 4-week-old, and 8-week-old mice, stained with toluidine blue. The expanded hypertrophic zones in 13del-MCDS mice are marked by the double heads and **i** quantified. **j** In situ hybridization for 13del-Col10a1-specific mRNAs and immunohistochemistry staining for 13del-ColX-specific proteins on the proximal tibia growth plate of P10 mice. **k** A diagram representation of the position of the *13del-Col10a1* (pWF380 specific riboprobe

We generated a mouse model for MCDS by homologous recombination of the endogenous *Col10a1* locus with the 13 bp deletion (*c.1857_1869del13*) mutant allele in mouse ES cells (Fig. 1a). At birth, the gross appearance of heterozygous (M/+) and homozygous (M/M) mice for the *Col10a1-13del* (M) allele is comparable to that of wild-type (+/+) littermates, albeit smaller in appearance (Fig. S1a-d). Dwarfism and skeletal abnormalities become apparent by postnatal day 10 (P10) in an allele dosage-dependent manner (Fig. 1b) and are maintained throughout postnatal life (Fig. 1b). M/M mice exhibited a more severe dwarfism than M/+ littermates. This is consistent with shortening of the vertebral column (Fig. 1c) and vertebral bodies (Fig. S2a and b), and the shortening of long bones such as the femur (Fig. 1d) and tibia (Fig. 1e) that are more pronounced, resulting in a disproportionate dwarfism phenotype as reported in human cases.

Hand involvement has been reported in 47% (7/15) of cases in a study of MCDS patients.^24^ The changes consisted of shortening of the tubular bones and metaphyseal cupping of the proximal phalanges and metacarpals. The hind paws of P10 and 4-week-old mice were examined by histology and skeletal preparation. No obvious histomorphology changes can be observed in M/+ mice, but the metacarpals were shorter in M/M mice (Fig. S3a). The growth plates are expanded, and the distance between two growth plates in the same bone is diminished, consistent with retarded bone growth (Fig. S3a). At 4 weeks of age, shortening of phalanges and metacarpals was observed in both M/+ and M/M mice (Fig. S3b).

Bowing of tubular bones and flaring of metaphases are also radiographical features of human MCDS and were observed in M/+ and M/M mice. The radio-dense band in the X-ray images and bulged regions in the skeletal preparation samples indicate that flaring of the metaphyses was also exhibited in the costochondral joints (Fig. S4a & S4b) of the rib cage. In human MCDS, cupping, splaying, and/or sclerosis at the costochondral junctions are commonly observed.^25^ The thoraces of the M/+ and M/M mice were narrower (Fig. S4a), and the costal cartilage of the anterior rib cage was smaller (Fig. S4b), likely due to abnormal endochondral ossification in the costochondral joint (Fig. S4c).

*Coxa vara* is a reduction of the angle between the femoral shaft and the femoral neck. These angles were measured in 4-week-old animals. The averaged degrees were 139°, 121.3°, and 118.3° for +/+ (WT), M/+, and M/M mice, respectively (Fig. 1f, Fig. S5a), which are statistically significant (Fig. 1g). Given that an enlarged capital femoral epiphysis has been reported in human MCDS, we performed a histological examination. The femoral epiphysis of wildtype mice is a rounded structure with a ball-and-socket fit, allowing a smooth rotation of the acetabulum (Fig. S5b). In M/+ and M/M mice, the surface is slightly flattened (Fig. S5b). In addition, the articular cartilage thickness in the capital femoral epiphysis was thinner, but the growth cartilage region was expanded (Fig. S5c and S5d). Similar to other long bones, the femoral necks were shorter due to abnormal endochondral ossification. Moreover, the trabecular and cortical bone were thickened in the femoral neck, probably caused by the mechanical stress at this site, which correlated with the severity of the *coxa vara* (Fig. S5a). In MCDS patients, prolonged bed rest or immobilization of the lower limbs can temporarily normalize the irregular metaphyses seen on X-rays, indicating a mechanical contribution.^26,27^ This was also the case in the 13del-MCDS mice, where metaphyseal abnormalities improved when limb weight loading was reduced by unilateral sciatic neurectomy to immobilize the limb (Fig. S6). Thus, our model fully recapitulates the human MCDS characteristics.

Radiographic changes of the growth plates are well characterized in human MCDS patients. In addition, histological studies of patient iliac crest samples showed expansion of the hypertrophic zone as observed in the mouse.^14,15^ The tibia in M/+ and M/M mice is much shorter than that of controls, as we expected, with the growth plates displaying a dramatic expansion of the hypertrophic zone, illustrated from histological analyses at birth (postnatal P0) and 8 weeks of age (Fig. 1h). The height of the hypertrophic zone showed that at P0, only a slight expansion of the hypertrophic zone was observed in M/M mice, but not in M/+ mice (Fig. 1i). The expansion of the hypertrophic zone was most obvious at P10 for both M/+ and M/M mice in a dosage-dependent manner, coincident with shortening of the axial and appendicular skeletons. The biggest difference is at 4 weeks, where the relative expansion of the hypertrophic zone to WT littermates is 3.2- and 3.9-fold for M/+ and M/M mice, respectively. At 8 weeks, the height of the hypertrophic zone of M/+ was comparable to that of the wildtype mice, while there was still a 2.7-fold expansion in M/M mice (Fig. 1i). Overall, our 13del-MCDS mouse model recapitulated all the clinical features of MCDS (Table 1).

**Table 1:** Comparison of human MCDS patients and the 13del-MCDS mouse

Human MCDS Features	Reference	13del-MCDS mouse
Dwarfism	Warman et al. (1993)^2^	Yes (Fig. 1b)
Presentation of phenotypes in nonfamilial cases is no earlier than the second year of life	Lachman et al. (1988)^25^	Yes. Dwarfism phenotype was not obvious at P0 in M/+ mice (Fig. S1) and become noticeable by P10 (Fig. 1b)
Enlarged capital femoral epiphysis	Lachman et al. (1988)^25^	Yes (Fig. S5)
*Coxa vara*	Lachman et al. (1988)^25^	Yes (Fig. 1f, g)
Flaring of metaphysis	Lachman et al. (1988)^25^	Yes (Fig. 1e)
Short stature and bowed legs	Hasegawa et al. (1994)^77^	Yes (Fig. 1c, d)
Mild platyspondyly	Hasegawa et al. (1994)^77^	Yes (Fig. S2)
Shortening of the tubular bones and metaphyseal cupping of the proximal phalanges and metacarpals	Elliott et al. (2005)^24^	Yes (Fig. S3)
Anterior rib changes	Lachman et al. (1988)^25^	Yes (Fig. S4)
Growth plate changes	Richmond and Savarirayan (2019)^1^	Yes (Fig. 1h, i)

Next, to confirm that the expanded hypertrophic zone is related to the expression of the mutant *Col10a1*, we assessed the expression and localization of *Col10a1* at P10, when endochondral ossification appears most active. Here, we used a 13del-specific riboprobe and an antibody specific to the 13del mutated collagen X protein (Fig. 1j) and confirmed that the 13del (M) allele was specifically expressed by HCs, and the misfolded proteins were localized inside the cells in M/+ and M/M mice (Fig. 1k).

### Activation of ER stress correlates with the altered cell differentiation and cell-cycle re-entry of hypertrophic chondrocytes

**Fig. 2:**
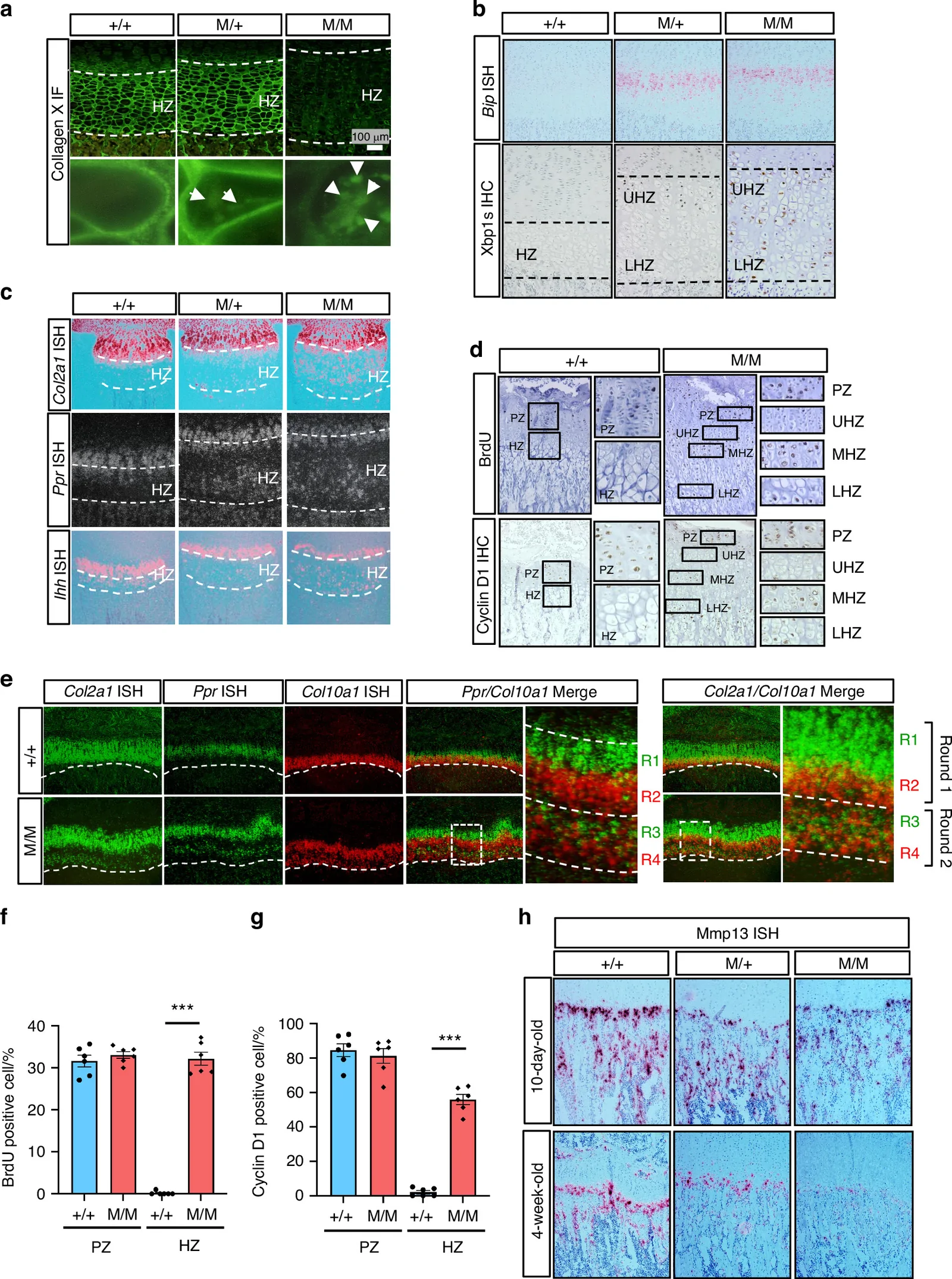
Changes in the hypertrophic chondrocytes of 13del-MCDS mice. **a** Immunofluorescent staining of CollagenX (ColX) was performed on the proximal tibia growth plate of P10 mice. ColX proteins can be detected in the ECM of +/+ and M/+ mice, while some or most are accumulated inside the cells in M/+ and M/M mice, respectively (white arrows). **b** In situ hybridization of Bip mRNA and immunohistochemistry staining of Xbp1s on the proximal tibia growth plate of 10-day-old mice. **c** In situ hybridization of *Col2a1*, *Ppr*, and *Ihh* mRNA on tibia paraffin sections on the proximal tibia growth plate of 10-day-old mice. **d** BrdU staining and Cyclin D1 immunohistochemistry staining on the proximal tibia growth plate of 4-week-old mice. Higher magnifications of the respective boxed regions in the proliferative zone and hypertrophic zone are shown (HZ, hypertrophic zone; UHZ, upper hypertrophic zone; MHZ, middle hypertrophic zone; LHZ, lower hypertrophic zone). **e** In situ hybridization of *Col2a1*, *Col10a1* and *Ppr* mRNA on proximal tibia growth plate of 4-week-old mice, showing two consecutive Col10a1 positive hypertrophic zones and two rounds (R1 and R2) chondrocyte hypertrophy. Quantification of positive cells for BrdU in (**f**) and Cyclin D1 in (**g**) (*n* = 3). **h** In situ hybridization of *Mmp13* mRNA on the proximal tibia growth plate of 10-day-old and 4-week-old mice

Collagen X is secreted and localized in the pericellular and extracellular matrices in wild-type (+/+) mice (Fig. 2a). As one of the *Col10a1* loci is normal in M/+ mice, collagen X was detected in the extracellular region, likely containing mostly normal collagen X, while the mutant proteins remained intracellular. Indeed, in M/M mice, most, if not all, collagen X (ColX-13del) proteins were intracellular in the HCs (Fig. 2a). The intracellular accumulation of ColX-13del proteins confirmed that they are misfolded and induce ER stress, as in previous reports.^14^ ER stress markers, such as CHOP, GADD34, and ATF3, were upregulated in HCs of growth plates in M/M mice (Fig. S7a, b, and c), as well as key markers, Bip and Xbp1s (Fig. 2b).

Activation of ER stress in HCs did not lead to increased apoptosis in P10 13del-MCDS mice (Fig. S8), but led to phenotypic changes of HCs, consistent with previous reports.^14^ HCs in the growth plate have been shown to be plastic under stressed conditions.^14^ HCs in M/M mice growth plates express *Col2a1* and pre-HC markers (*Ppr* and *Ihh*) at P10 (Fig. 2c), while changes in newborn mice are less obvious (Fig. S9). At P10, p57^Kip2^ is expressed in the majority of cells within the hypertrophic zone of M/M mice (Fig. S10) and serves as a marker for proliferating chondrocytes that have exited the cell cycle,^28,29^ and consistent with previous report for the 13del-tg mice (Tsang et al. ^14^), suggesting that HCs in M/M mice have exited the cell cycle.

At 4 weeks old, cyclin D1, a marker for cells in the G1 phase, was markedly reduced in the upper hypertrophic chondrocyte layer both in +/+ and M/M mice, concomitant with cell-cycle exit at this stage (Fig. 2d, g). However, cyclin D1 was re-expressed in the middle and lower hypertrophic zones in M/M mice (Fig. 2d, g), indicating re-entry into the proliferative phase. Further, BrdU incorporation, largely absent in the upper hypertrophic layer, was detected in the middle and lower hypertrophic layers in M/M mice (Fig. 2d, f), indicating progression to S-phase in a subset of cells.

Together, these findings suggest that proliferating chondrocytes in M/M mice initially exited the cell cycle, underwent hypertrophic differentiation, and subsequently re-expressed proliferative and pre-hypertrophic markers upon activation of the UPR, including *Ihh, Ppr*, cyclin D1, and BrdU incorporation. At 4 weeks of age, the M/M growth plate displayed a duplicated zonal organization of *Col2a1*-, *Ppr*-, and *Col10a1*-expressing domains, associated with ectopic proliferative activity within the expanded hypertrophic zone. This pattern is consistent with altered or repeated progression through chondrocyte differentiation states. (Fig. 2e).

Consequently, with such a disruption in the endochondral ossification process, remodeling of the hypertrophic cartilage would be disrupted as well, evident by reduced expression of *Mmp13* at the chondro-osseous junction for 4-week-old M/+ mice, and barely detectable in M/M mice (Fig. 2h). Based on the phenotypic analyses and further investigation of the molecular changes associated with the cellular outcome, the remaining assessments will focus on comparisons between M/M and +/+ (WT) littermates.

### Involvement of the hypoxic pathway in hypertrophic chondrocytes under ER-stress

**Fig. 3:**
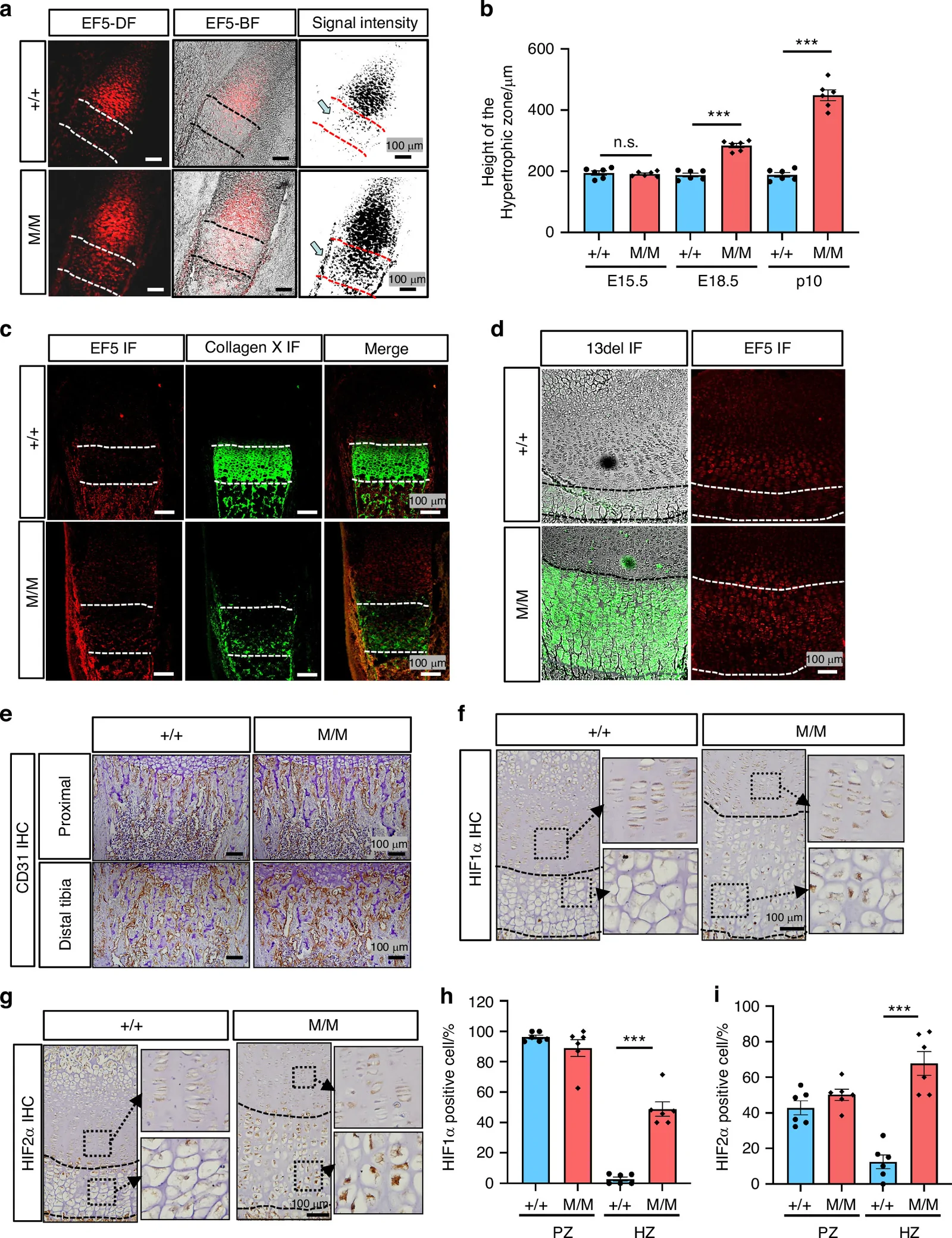
Hypoxia and activation of the HIF pathway in the growth plate of 13del-MCDS mice. **a** EF5 staining on the proximal tibia growth plate of +/+ and M/M mice at E15.5, in EF5-Cy3 dark field (EF5-DF), overlapped with the bright field image (EF5-BF), and with the signal intensity transferred by ImageJ (Signal intensity), with dotted lines marking the hypertrophic zone. **b** Measurement of the height of the hypertrophic zones at the midsection of the proximal tibia growth plates from prenatal (E15.5, E18.5) to postnatal P10, with three sections from each biological sample (*n* = 9). **c** EF5 staining and the corresponding immunofluorescence staining with Collagen X or 13del-specific antibodies for +/+ and M/M mice, respectively, on the proximal tibia growth plate at E18.5. **d** EF5 staining and the corresponding immunofluorescence staining with 13del-specific antibodies of +/+ and M/M mice proximal tibia growth plate P10. **e** Immunohistochemistry staining of CD31 on 10-day-old proximal tibia growth plate, focusing on chondro-osseous junction and the trabecular bone region. **f**, **g** Immunohistochemistry staining for HIF1α and HIF2α proteins on 10-day-old proximal tibia growth plate. Higher magnifications of the boxed areas in the proliferative and hypertrophic regions are shown on the right. Dotted lines mark the hypertrophic zones, and the percentage of positive cells in each zone was quantified for HIF1α (*n* = 6) (**h**) and HIF2α (*n* = 6) (**i**). Scale bar = 100 μm (panels **a**–**g**)

The fetal growth plate cartilage is an avascular tissue, and cells located away from the peripheral tissues and the chondral-osseous junction experience a lower oxygen concentration, triggering a hypoxic response.^30^ Thus, expansion of the hypertrophic zone in 13del-MCDS mice may trigger an elevated hypoxic response in the upper region of the hypertrophic zone, due to a further distance from the blood vessel invasion area. To test this hypothesis, we used a metabolic marker of hypoxia (EF5) to detect a change in oxygen level in vivo. First, we studied hypoxia of the developing proximal tibia growth plate region at E15.5, where an intense hypoxic region is detected at the central area of the proliferating zone and upper hypertrophic zone of the growth plate cartilage in wild-type mice (Fig. 3a), consistent with a previous report.^30^ In M/M mice, a strong signal is also detected in the hypertrophic zone. However, at this stage of development, the hypertrophic zone of the M/M mice is not expanded (Fig. 3b) compared to the +/+ mice.

Next, we performed a similar analysis on the E18.5 tibia growth plate, where a mild but significant expansion of the hypertrophic zone can be detected (Fig. 3b). Co-immunofluorescence staining of EF5 and Collagen X in +/+ mice showed mild staining of EF5 in the upper Collagen X-localizing region corresponding to the pre-hypertrophic zone (Fig. 3c). However, in M/M mice, EF5 staining is most obvious towards the middle and lower regions of the hypertrophic zone, corresponding to the intracellular localization of ColX-13del proteins, but an area not likely to be deprived of a supply of oxygen, as it is closest to the marrow space (Fig. 3c). Together with the observation at E15.5, these results suggest that the hypoxia in M/M HCs may not be related to the hypertrophic zone expansion.

At P10, a stage of active bone growth, the hypertrophic zone in +/+ mice is the highest and the most significant expansion in M/M mice (Fig. 3b). Immunofluorescence staining of the 13del Collagen X protein is localized to the full region of the hypertrophic zone specific to M/M mice (Fig. 3d). At this stage, EF5 staining is more prominent towards the upper hypertrophic region of the proximal (Fig. 3d) and distal tibia growth plates (Fig. S11), different from the prenatal growth plate, indicating the cells at the lower hypertrophic zone have changed, perhaps adapted to the initial stress signals. So far, the EF5 data point to an intrinsic, cell-autonomous effect, although we cannot completely exclude a contribution from lower exogenous oxygen levels due to the expansion. However, staining for PECAM-1 (CD31) at P10 showed that signal intensity and distribution in the tibia trabecular bone regions are very similar compared to +/+ littermates with no observable differences (Fig. 3e).

Hypoxia is associated with the activation of *HIF1α* and *HIF2α*.^31^ Immunohistochemistry staining showed an accumulation of HIF1α and HIF2α proteins in the reserve and proliferative zones, but not in the hypertrophic zone in +/+ mice (Fig. 3f, g). However, in M/M mice, there is ectopic expression of HIF1α and HIF2α across the whole region of the expanded hypertrophic zone (Fig. 3f, g). Quantification of the number of HIF1α- and HIF2α-positive cells confirmed the specific change in the hypertrophic region (Fig. 3h, i). The expression and/or stabilization of both HIF proteins is maintained throughout the hypertrophic zone.

To test whether HIF1α functionally contributes to the 13del-MCDS growth plate phenotype, we attempted to conditionally inactivate Hif1α in Col10a1-expressing hypertrophic chondrocytes,^20,30^ However, HIF1α protein remained detectable in M/Col10a1Cre; Hif1α^F/F^ growth plates, indicating incomplete functional inactivation. Thus, we could not establish a direct relationship between Hif1a and the observed cellular changes. We provide the outcome of this test in the Supplementary Information to document the limitation of this genetic approach (Fig. S12a–d).

### Activation of hypoxic response in hypertrophic chondrocytes of 13del-MCDS mice is an intrinsic response to ER stress

Data so far support an intrinsic response of HCs expressing *13del-Col10a1*. In M/M mice, given that most, if not all, 13del Collagen X proteins are retained within the HCs, with no normal Collagen X in the extracellular matrix, effectively a *Col10a1*-null outcome. To exclude contribution from changes to the pericellular matrix environment,^32^ inducing a hypoxic response, we assessed EF5 staining in *Col10a1*-null mice^33^ that showed no signal changes in HCs compared with WT mice (Fig. S13).

Next, we studied the Talus bone in the same way. Talus is the largest of the tarsal bones, located between the distal tibia and calcaneum (Fig. S14a and b). It does not have a growth plate but contains HCs through a process like secondary ossification (Fig. S14c), expresses *Col10a1* (Fig. S14d), and deposits a Collagen X-containing matrix (Fig. S14e). In 10-day-old +/+ Talus bone, EF5 signal is absent, but is stained with high intensity in M/M Talus, coinciding with the intracellular accumulation of ColX-13del proteins (Fig. S14f). Again, this is unlikely due to structural change, as the Talus bones are of similar size between +/+ and M/M mice, and no EF5 staining is observed for this bone in *Col10a1*-null mice as well (Fig. S14g).

**Fig. 4:**
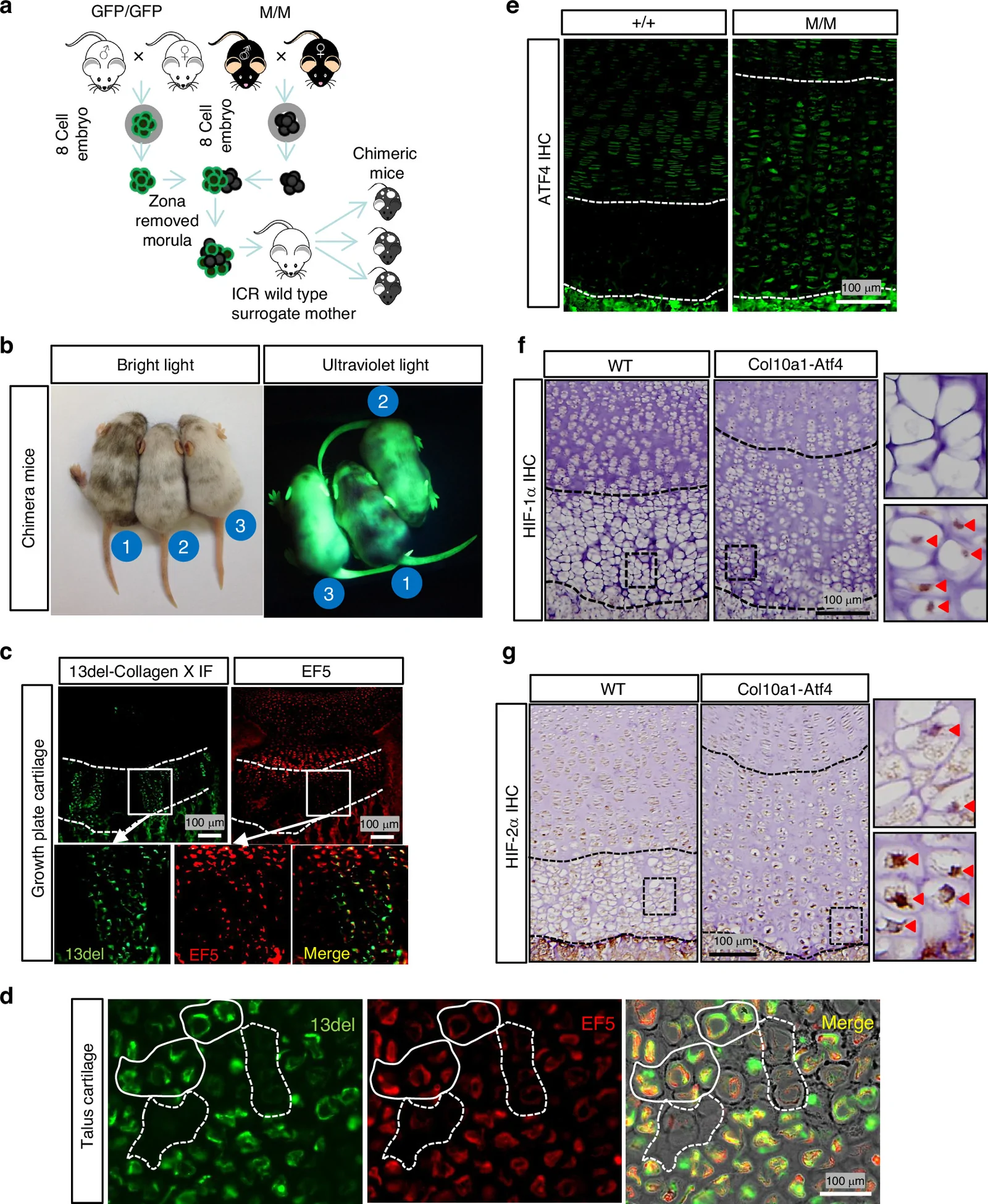
ER stress increases cell-autonomous oxygen demand and ATF4 stabilizes Hif1a in hypertrophic chondrocytes. **a** Scheme illustrating the generation of GFP/MCDS chimeric mice by morula-morula aggregation derived from WT-GFP and M/M mice. **b** Three chimera mice with differing chimerism, evident by the patchy black and white coat color and varying GFP fluorescence under UV lighting at P9. **c** Immunofluorescence staining with columns of cells with intracellular accumulation of 13del-ColX proteins, corresponding to EF5-positive HCs in the P9 proximal tibia growth plate. Higher magnification of the boxed column is shown, and the merged panel highlights the persistence of EF5 staining in HCs with 13del-ColX proteins. Scale bar = 100 μm. **d** EF5 and 13del-ColX co-staining in Talus bone showing a “salt and pepper” distribution of chimeric HCs. Solid lines circle clusters of M/M cells and dotted lines circle +/+ cells, with EF5 staining restricted to M/M cells. Scale bar = 50 μm. **e** Immunofluorescence staining with ATF4 antibody of +/+ and M/M P10 mice proximal tibia growth plate. Dotted lines indicate the hypertrophic zone. Scale bar = 100 μm. Immunohistochemistry staining for HIF1α (**e**) and HIF2α (**f**) at P10 of the proximal tibia growth plate of transgenic mice (*C10-Atf4*) over-expressing *Atf4* in HCs. Higher magnification of a hypertrophic region highlights the maintenance of stabilization of HIF proteins by the ectopic expression of *Atf4*. Scale bar = 100 μm

To distinguish between cell autonomous and non-cell autonomous effects, we generated mouse chimeras by morulae aggregation containing a mixture of M/M (black coat) and WT (GFP-expressing, white coat) cells (Fig. 4a). The degree of chimerism can be visualized by the different coat color as well as the green fluorescent protein (Fig. 4b). In addition, M/M derived HCs can be identified by the accumulation of ColX-13del proteins using the specific antibody, as indicated by the columns of ColX-13del-specific HCs in the P10 tibia proximal growth plate (Fig. 4c). Significantly, administration of EF5 to mark cells under a hypoxic state, in addition to the expected signal in the proliferative zone, EF5 signals are detected continuing into the columns of M/M HCs, and not in WT chondrocytes, even for WT HCs adjacent to the M/M HCs (Fig. 4c). To further confirm this intrinsic effect of the M/M HCs, we assessed the relationship in the Talus bone containing a salt-and-pepper mixture of WT and M/M HCs and showed that EF5 signals are restricted to groups of M/M HCs (Fig. 4d, solid line regions) but not in WT HCs (Fig. 4d, dotted line regions).

Previously, we reported that ATF4 upregulation in HCs of 13del-Tg mice was part of UPR activation.^20^ To see whether a similar change occurs in M/M mice, we performed ATF4 immunostaining in the growth plate. ATF4 expression was markedly increased in HCs of M/M mice compared to controls (Fig. 4e), indicating differential ATF4 expression in the M/M growth plate. We then tested whether ATF4 activation relates to hypoxia signaling changes. We previously showed that ectopic *Atf4* activation in HCs of a transgenic mouse (C10-Atf4) partly recapitulated the 13del-Tg growth plate features, with hypertrophic zone expansion and reactivation of *Sox9* and other chondrogenic markers such as *Col2a1*.^20^ Using the *C10-Atf4* transgenic mouse, we showed that both HIF1a and HIF2a were activated in HCs concomitant with the ectopic activation of ATF4 (Fig. 4f, g). Together, the findings support an intrinsic effect from the expression of the Col10a1-13del, activating the ER-stress in which the PERK/ATF4 axis is linked to activation of the HIF proteins, mimicking a hypoxic response as part of the survival and programming process.

### p53/p63 network consolidates survival and progression toward cell-cycle re-entry

**Fig. 5:**
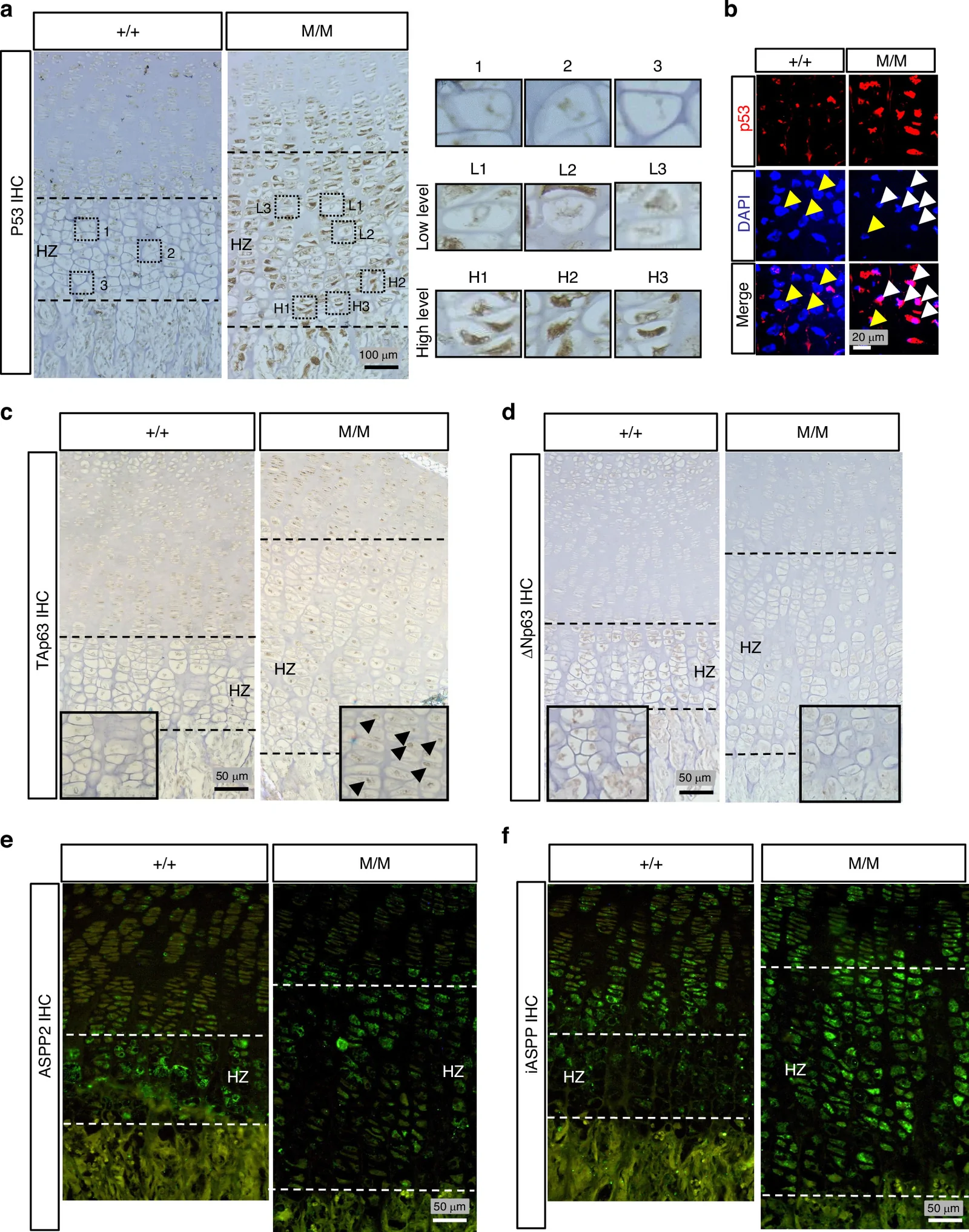
Dynamic changes in p53/p63 and regulators, ASPP2 and iASPP in 13del-MCDS mice hypertrophic chondrocytes. **a** Immunohistochemistry staining of p53 on the 10-day-old proximal tibia growth plate. Specific regions of the hypertrophic zone (HZ) in +/+ and M/M mice are shown in higher magnification, illustrating very low staining in +/+ in regions 1, 2, and 3. Whereas in M/M mice, there are varying low (L1, L2, and L3) and high (H1, H2, and H3) p53-expressing regions. **b** Co-immunofluorescence staining of p53 and DAPI showing p53 in +/+ HCs is cytoplasmic, whereas in M/M mice, p53 is primarily localized to the nucleus (yellow arrowheads). **c** Immunohistochemistry staining of Tap63 showed an upregulation in M/M HCs (black arrowheads). **d** Immunohistochemistry staining of ΔNp63 showed a downregulation in M/M HCs. **e** Immunofluorescence staining of ASPP2 and **f** iASPP proteins on 10-day-old proximal tibia growth plates, showing a differential and dynamic down- and up-regulation in HCs of M/M mice, respectively. Scale bar = 50 μm

p53 is activated in cellular stress responses, including ERSS, and is one of the earliest transcription factors activated in the HCs of 13del-Tg mice.^14^ p53 is considered a tumor suppressor with multiple roles in regulating the cell cycle and apoptosis. In the tibia proximal growth plate of P10 +/+ mice, p53 proteins are detected mainly in proliferative and preHCs but little in HCs (Fig. 5a). In contrast, cells with a high level of p53 are detected in the expanded hypertrophic zone of M/M mice (Fig. 5a). The level of p53 protein in HCs correlated with the cell morphology in the M/M hypertrophic zone. M/M HCs with low p53 levels exhibited a relatively normal large and round morphology (Fig. 5a L1-L3), similar to +/+ HCs (Figs. 5a, 1–3), and those with high p53 levels were smaller and flattened, which are more pre-HC-like (Fig. 5a, H1-H3) with nuclear localization of p53 within more condensed nuclei (Fig. 5b). Interestingly, TAp63, a p53 homolog that is also inducible by cellular stress, with similar functions in cell cycle control and apoptosis, showed similar expression patterns with ectopic expression in M/M HCs (Fig. 5c).

In contrast, ΔN-p63, a negative regulator of TAp63, is normally expressed in +/+ HCs, but is down-regulated in M/M HCs (Fig. 5d). Upon cellular stress, ΔN-p63 is known to be downregulated by transcriptional control and protein degradation pathways to facilitate the actions of TAp63.^34^ In summary, p53 and TAp63 were upregulated while ΔNp63 was downregulated upon ERSS activation. Surprisingly, activation or stabilization of nuclear p53/TAp63 did not lead to robust apoptosis of M/M HCs, indicating a targeted anti-apoptotic mechanism could be involved.

The apoptosis-stimulating proteins of the p53 (ASPP) family of proteins are known modulators of p53-apoptotic function. ASPP1 and 2 enhance the pro-apoptotic function of p53/TAp63, while iASPP has an antagonistic function to ASPP1 and ASPP2, which inhibits p53-mediated cell death.^35^ In P10 +/+ tibia proximal growth plate, ASPP2 was predominantly expressed and localized in the hypertrophic zone (Fig. 5e), whereas iASPP was mainly detected in the lower proliferative and pre-HCs. In M/M mice, iASPP was highly expressed in HCs while ASPP2 was downregulated (Fig. 5f), suggesting a protective role against p53-mediated apoptosis, and p53/TAp63 may only exert a cell-cycle controlling function in this context.

### Clearance of unfolded ColX-13del via autophagy correlates with survival of hypertrophic chondrocytes and cell-cycle reentry

**Fig. 6:**
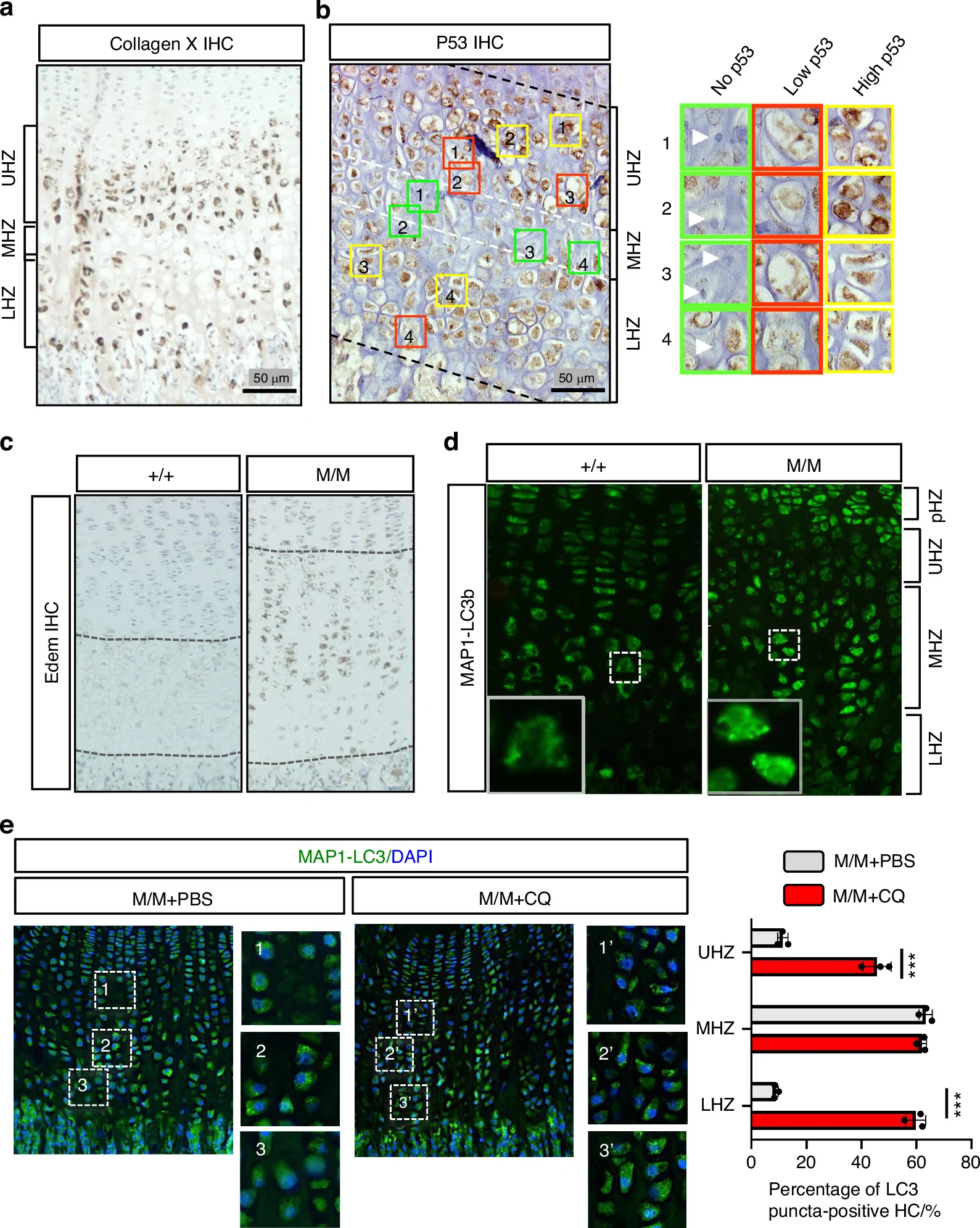
Protein clearance is correlated with the hypertrophic chondrocyte differentiation status. **a** Immunohistochemistry staining of Collagen type X on paraffin sections of the tibia proximal growth plate from 4-week-old M/M mice. Scale bar = 50 μm. **b** Immunohistochemistry staining of p53 on paraffin sections of the tibia proximal growth plate from 4-week-old M/M mice. Scale bar = 50 μm. **c** Immunohistochemistry staining of Edem on paraffin sections of the tibia proximal growth plate from P10 M/M mice. **d** Immunofluorescence staining of Map1-lc3b on paraffin sections of the tibia proximal growth plate from 4-week-old M/M mice and their littermate controls. Boxed regions are the amplified dotted area. **e** Chloroquine was administered to M/M mice to monitor the dynamics of autophagy activity by blocking the fusion of autophagosomes with lysosomes. Immunofluorescence staining and quantification of LC3 puncta cells demonstrated an increase in LC3 puncta-positive cells in the UHZ and LHZ of MCDS mice after the chloroquine administration. UHZ (1, 1’): the upper hypertrophic zone; MHZ (2, 2’): the middle hypertrophic zone; LHZ (3, 3’): the lower hypertrophic zone

Localization of p53 proteins showed a very similar pattern to that of ColX-13del protein in the 4-week-old M/M hypertrophic zone. There was an upregulation in both the upper hypertrophic zone and lower hypertrophic zone, but it was downregulated in the middle hypertrophic zone (Fig. 6a, b). Consistently, cells with high p53 protein levels in both the upper hypertrophic zone and lower hypertrophic zone were smaller chondrocytes, and those with low p53 levels had normal HC morphology (Fig. 6b).

Interestingly, a population of cells with almost no p53 expression was detected in the region where intracellular collagen X was cleared (Fig. 6a, b). Therefore, we proposed that in this context, the protein clearance mechanism, likely autophagy, is also activated under the UPR, hypoxia, and p53/63 pathways as an outcome to alleviate HC stress in M/M mice. EDEM, a target of XBP1s, is involved in ER-associated degradation (ERAD) for the removal of unfolded protein from cells and is upregulated in the middle hypertrophic zone and lower hypertrophic zone (Fig. 6c), suggesting activation of ER-associated degradation. Since proteasome activity was not directly measured, we interpret these findings as evidence that multiple protein-quality-control pathways are engaged in response to mutant collagen X accumulation.

Immunofluorescence staining of Map1lc3 (LC3) was used to monitor autophagy activity. Expectedly, puncta staining of LC3 was observed in the hypertrophic zone of the M/M growth plate (Fig. 6d). The presence of puncta staining of LC3 indicates the processing to LC3II localized to the membranes of autophagosomes.^36^ The region of high autophagy activity correlated with the region of cell cycle re-entry in M/M HCs, which is predominantly located in the middle hypertrophic zone (Fig. 6d). LC3 immunofluorescence staining and quantification revealed puncta staining that was increased in the M/M middle hypertrophic zone, indicating engagement of autophagy-associated pathways (Fig. 6e, zone 2). Following Chloroquine treatment, LC3 puncta accumulation was increased in the upper (zone 1’) and lower (zone 3’) hypertrophic zone (Fig. 6e), consistent with ongoing autophagy-associated flux. These findings indicate that autophagy-associated protein-quality-control mechanisms are activated in mutant hypertrophic chondrocytes under ER stress.

## Discussion

Five genetically modified mouse models (three transgenic and two gene-targeted) for MCDS have been previously reported. FCdel-Tg and 13del-Tg are transgenic mice carrying human *COL10A1* C-terminal truncation (*p.P620fsX621*)^15^ and 13 bp frameshift (*c.1857_1869del13*) mutations,^14^ respectively. Whereas the *Cog* transgenic mouse expresses a mutant form of thyroglobulin under the control of the *Col10a1* promoter.^37^ There are two gene-targeted mouse models with human- equivalent mutations, p.PN617K^22^ and p.Y632X,^23^ in the mouse *Col10a1* locus. All these mice exhibited some features of human MCDS but were not characterized for the full spectrum of human MCDS characteristics. Here, we generated and characterized a gene-targeted mouse model that introduces the same 13-bp deletion as in the 13del-Tg mouse, at the endogenous *Col10a1* locus. Heterozygous (M/+) and homozygous (M/M) 13del-MCDS mice phenocopied all reported characteristics of human MCDS (Table 1). It is noted that in human patients, skeletal changes develop with weight bearing at 3–5 years of age, at which time bowing of the legs becomes apparent. This new MCDS mouse model presents as an excellent preclinical model to assess therapeutic options.

Activation of the UPR as part of the ERSS has been shown to be the pathological mechanism of MCDS, shifting the cell state for survival.^14,20,22^ We confirmed all aspects of UPR activation and adaptation in the newly generated 13del-MCDS mice. As in other models, this adaptive response derailed terminal differentiation and ossification, resulting in reduced growth and the characteristic dwarfism. While the core UPR circuitry has been investigated, the mutation- and tissue-specific mechanisms that govern chronic survival of the HCs versus pathology in the growth plate remain to be defined, as does the potential to introduce safe and targeted ways to tune these pathways for therapy. Here, we provide further insights into how hypertrophic chondrocytes adapt to chronic ER stress, leading to a disruption of endochondral ossification. Our findings reveal coordinated activation of multiple stress-response pathways and protein-quality-control mechanisms that are spatially associated with hypertrophic chondrocyte survival, altered differentiation dynamics, and ectopic proliferative activity within the expanded hypertrophic zone, suggesting that these pathways may contribute to the adaptive response of mutant hypertrophic chondrocytes.

The canonical UPR pathways are all upregulated in MCDS HCs, with *Chop* (*Ddit3*) amongst the highest levels among all the upregulated genes. CHOP is well known to promote ER stress-induced apoptosis;^38,39^ however, both this study and previous reports show that this does not lead to increased apoptosis in MCDS HCs. Indeed, in the context of the 13del-Tg mice, CHOP offered a pro-survival role, as ablating of *Ddit3* led to increased apoptosis,^20^ suggesting that other protective pathways are involved. The mechanism for survival may further involve the cooperation between ATF4/CHOP in the induction of *Fgf21* under ER stress, which appears to occur in a cell-autonomous manner.^20^ *Fgf21* has also been shown to be upregulated in other MCDS mouse models.^40^ FGF21 signaling is mediated by FGFR1, FGFR2, or FGFR3 in conjunction with the co-regulator β-klotho,^41^ and FGF21 is a well-recognized metabolic regulator.^42^

Previously, we proposed that HCs in 13del-Tg mice survive by a mechanism whereby changes in ER-mitochondria communication reduce import of calcium coupled to maintenance of mitochondrial membrane polarity and glucose uptake.^43^ This provides the ATP supply required to preserve the energy balance necessary for cell survival under ER stress. ER stress and hypoxia are tightly linked. Hypoxia can induce ER stress and activate the UPR, as seen in solid tumors and ischemic tissues, where low oxygen levels disrupt ER protein folding, leading to misfolded-protein accumulation and activating PERK, IRE1α, and ATF6 signaling to promote cell survival.^44,45^ The PERK–eIF2α–ATF4 axis enhances cellular tolerance to hypoxic stress.^46^ Conversely, our findings suggest the reverse mechanism in HCs, where ER stress can itself drive hypoxia and activate HIF signaling in vivo.

Mechanistically, ER stress can promote mitochondrial respiration and cellular oxygen consumption. When ER protein-folding capacity is overwhelmed, such as during intense collagen synthesis in HCs, excess disulfide-bonding can raise oxygen consumption and the generation of reactive oxygen species (ROS).^47^ ER stress also increases Ca²⁺ transfer from the ER to mitochondria, stimulating oxidative phosphorylation (OXPHOS) and further increasing mitochondrial respiratory activity and oxygen utilization.^48,49^ Additionally, ER stress signaling as a survival cue can improve mitochondrial respiratory efficiency by promoting the assembly of respiratory chain supercomplexes and optimizing mitochondrial structural organization.^50,51^ Consistent with these mechanisms, our in vivo data indicate that ER stress in HCs can generate a cell-autonomous hypoxic state, likely driven by altered metabolism and increased oxygen consumption from misfolded collagen accumulation and ER overload. However, whether cell survival, cell-cycle re-entry, or reprogramming of M/M HCs are directly related to the activation of HIF proteins will require further substantiation, as the inactivation of *Hif1a* in HCs was inconclusive.

Within this framework, the PERK–ATF4 arm of the UPR appears to coordinate transcriptional, metabolic, and mitochondrial adaptations. ATF4 maintains mitochondrial respiratory capacity during ER stress by supporting OXPHOS protein expression and mitochondrial function,^52^ and it promotes HIF1α accumulation and stability under chronic hypoxia conditions.^53^ ATF4 is regulated at the translational level under hypoxia, partly through the UPR, linking ER stress signaling to hypoxia pathways,^54^ and it also enhances HIF1α in hypoxic osteoblasts.^55^ Although our data favor ATF4 acting upstream of HIF in this study, the relationship between ATF4 and HIF signaling may be context-dependent. Overall, ATF4-dependent metabolic reprogramming increases oxygen demand while stabilizing HIF-α signaling, creating a cell-autonomous hypoxic milieu and enabling a switch between mitochondrial respiration and HIF1α-driven glycolysis,^30,56^ which is essential for chondrocyte survival and function in the growth plate.

ER stress can induce the activation of p53, and hypoxia promotes the accumulation of p53 proteins.^57–60^ Importantly, nuclear accumulation of p53 can induce autophagy by activating autophagy-related genes and inhibiting mTOR signaling,^61,62^ linking stress-induced p53 activation to adaptive autophagic responses. Previously, in 13del-Tg mice, p53 was localized to the cytoplasm as part of a survival strategy under ER stress.^12,14,15^ In the context of 13del-MCDS, this response is more complex, as HCs with both low and high levels of p53 expression are observed at the postnatal stage of P10 and exhibit distinct morphologies. In cells with low p53 expression, p53 is predominantly localized in the cytoplasm, a pattern more consistent with its distribution in the rounded HCs of the upper hypertrophic region. In contrast, in cells with high levels of p53 expression, p53 is mainly localized in the nucleus, and the cells show a morphology resembling pre-hypertrophic chondrocytes in the lower hypertrophic region expressing *Ppr* and *Ihh*. Given that TAp63 is also upregulated and has functions similar to p53 in cell-cycle regulation and apoptosis induction,^63,64^ while its negative regulator ∆Np63 is downregulated, additional mechanisms must exist to counterbalance these pro-apoptotic and cell-cycle regulatory signals.

ASPP1/2 and iASPP are expressed in response to ER stress.^65,66^ Their expression is part of the broader cellular response to ER stress, in which they contribute to the complex signaling network that regulates p53-dependent decisions between cell survival and death, and they are also implicated in tumor suppression. ASPP2 enhances p53 binding to pro-apoptotic promoters, whereas iASPP inhibits the apoptotic transcriptional activity of p53 family members.^67^ Therefore, the downregulation of ASPP2 in 13del-MCDS HCs, together with the upregulation of iASPP, may provide an overall protective effect that promotes cell survival.

A novel finding in the 13del-MCDS mice is the reprogramming of HCs toward a proliferative state, followed by a second hypertrophic phase characterized by *Col10a1* expression in the 4-week-old growth plate. This was evident by the multiple layers of BrdU- and cyclin D1-positive cells. The mechanism underlying this phenotype in this context is unclear. For the reprogramming of stressed HCs, we have shown previously that this is due in part to the activation of ATF4 under ER-stress, which transactivates the expression of *Sox9*, a master transcription factor for chondrogenesis.^20^ For cell-cycle re-entry and additional rounds of chondrocyte differentiation, we noted a strong association with activation of autophagy and clearance of unfolded Collagen X, as well as changes in p53 signaling, as part of the survival strategy. Normally, as proliferative chondrocytes enter the hypertrophy program in the cartilage growth plate, they exit the cell cycle expressing *p57*^*kip2*^ (*Cdkn1C*),^14^ a cyclin-dependent kinase inhibitor that acts as a negative regulator of cell proliferation by blocking the G1/S transition of the cell cycle.^68^ p57^Kip2^ can function in parallel with the p53 pathway to induce cell-cycle arrest.^69^ Notably, a small subset of HCs lacked detectable p57^Kip2^ staining; thus, we cannot fully exclude the possibility that a minor population of proliferating chondrocytes failed to exit the cell cycle. In the 4-week-old growth plate of 13del-MCDS mice, following the first round of hypertrophy in the upper hypertrophic zone, a region in the middle hypertrophic zone shows little or no intracellular accumulation of Collagen X. This is accompanied by reduced p53 staining and is associated with activation of autophagy, as indicated by the Map1lc3-positive autophagosomes. Thus, clearance or reduction of p53 may help relieve inhibition of the G1/S transition and facilitate cell-cycle progression.

Here, we provide novel insights into the molecular pathogenesis of MDCS, as well as the context-dependent interplay between ER stress and the hypoxic response in the survival strategy of HCs expressing unfolded mutant Collagen X. Although all three UPR sensors (IRE1, ATF6 and PERK) are involved, the PERK-ATF4 axis appears to be a key sensor as part of the integrated stress response (ISR).^70,71^ This strategy involves the immediate management of unfolded protein for cell survival, within the specific context of hypertrophic chondrocytes in the cartilage growth plate undergoing endochondral ossification.

**Fig. 7:**
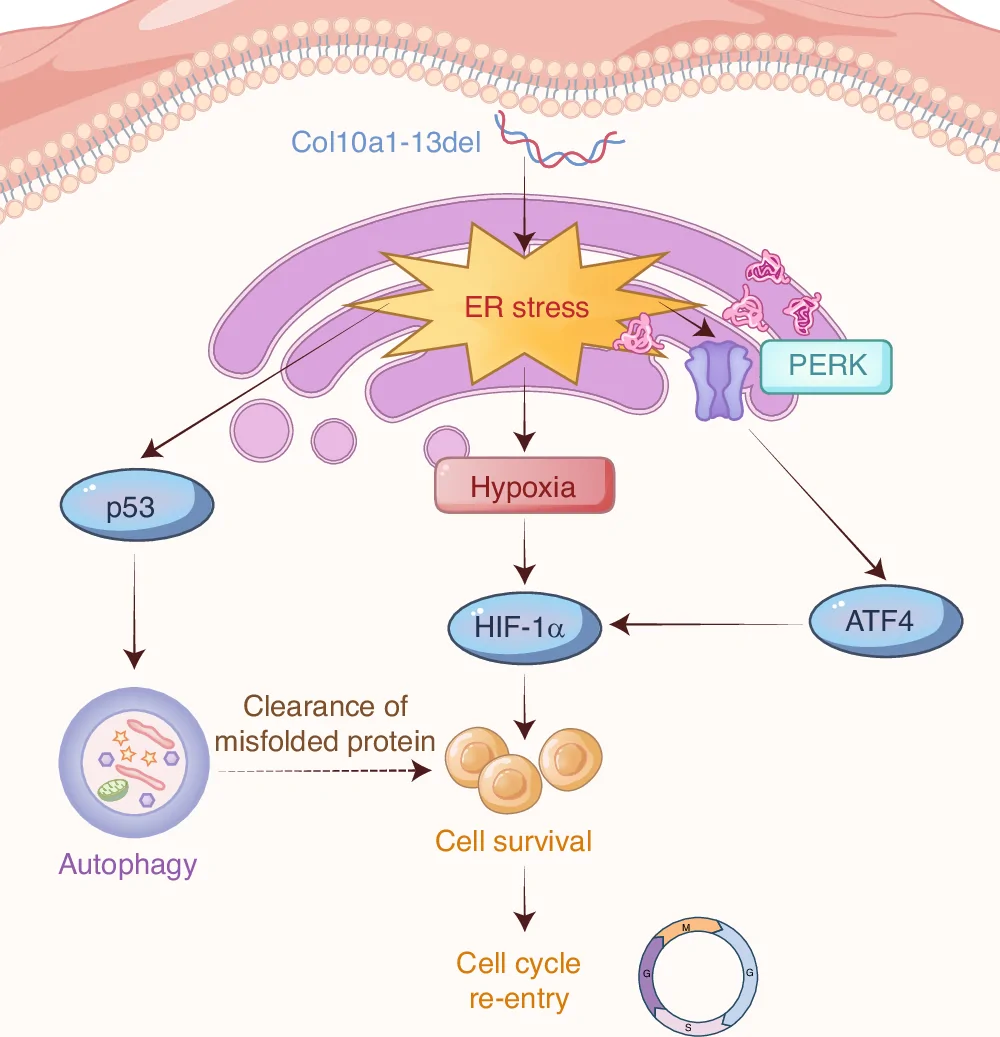
Proposed mechanism linking ER stress to integrated survival signaling in 13del-MCDS hypertrophic chondrocytes. A 13-bp deletion in *Col10a1* leads to production of misfolded collagen X and its accumulation within the endoplasmic reticulum (ER) of HCs, triggering ER stress. ER stress activates ATF4 and hypoxia/HIF signaling as an intrinsic adaptive response. In parallel, p53 signaling and autophagy are engaged. Autophagy facilitates the clearance of accumulated misfolded proteins, thereby reducing proteotoxic stress. Collectively, HIF, ATF4, p53, and autophagy form an integrated survival network that promotes HC survival, supports metabolic adaptation, and enables cell-cycle re-entry. This coordinated response enables partial adaptation to ER stress and contributes to the growth plate phenotype observed in 13del-MCDS mice

In summary (Fig. 7), HC survival in 13del-MCDS mice appears to involve the management of energy demands and stabilization of existing HIFα proteins. Although ERAD is activated throughout the hypertrophic zone, autophagy-associated mechanisms may contribute to more complete clearance of unfolded proteins, possibly via ATF4, which can induce autophagy under hypoxic conditions.^72^ In this context, reduced p53 levels may facilitate cell-cycle reentry in reprogrammed chondrocytes, potentially linked to Sox9 expression through ATF4-mediated transactivation. Finally, our findings support current therapeutic strategies targeting protein clearance using carbamazepine (CBZ)^23^ and the integrated stress response inhibitor (ISRIB), which targets the PERK-ATF4 sensor.^20^ A combination of these approaches, together with strategies to manage hypoxia-associated and energy- demand responses, may help reset the derailment of the endochondral ossification program and restore bone growth in MCDS patients.

## Methods and materials

### Generation of 13del-MCDS mice

All mouse work is approved by the Committee on the Use of Live Animals in Teaching and Research (CULATR) of the University of Hong Kong. The 13 bp deletion was introduced in the exon 3 of the *Col10a1* locus in the targeting construct (Fig. 1a). In addition, the polyA and pGK-neo (flanked by Frt sites; for ES cell G418 selection) sequences were inserted in the same exon. 13del-MCDS mice were generated by standard homologous recombination in ES cells (from 129svev mice). The targeted ES cells were injected into blastocysts (from C57bl/6). The germline-transmitted mice were crossed with Flp mice^73^ to remove the Flp-flanked pGK-neo sequence. Mutant mice can be identified by 13del-MCDS forward and reverse PCR primers, which flank the 13 bp deletion site, and a 13 bp size difference of the PCR product can be shown in a 3% (w/v) agarose gel electrophoresis (Table 2). The 13del-MCDS mouse line has been bred as heterozygous (M/+) x heterozygous (M/+), and wild type (+/+) and homozygous (M/M) in the offspring are used as controls and mutants.

**Table 2:** PCR primer oligonucleotides

Reagent	Sequence
13del-MCDS-Forward:	5’-CCCAGGCATATACTATTTCTC-3’
13del-MCDS-Reserse:	5’-TAGCCTTTGCTGTACTCATC-3’
HIF-1a-Flox-Forward:	5’-CGTGTGAGAAAACTTCTGGATG-3’
HIF-1a-Flox-Reverse:	5’-AAAAGTATTGTGTTGGGGCAGT-3’
HIF1a-recombination-Forward:	5’-TGGGGATGAAAACATCTGCTTTG-3’
HIF-1a-recombination-Reverse:	5’-AAAAGTATTGTGTTGGGGCAGT-3’
ColX-Cre-Forward:	5’-TCAGTCGTAGAACATCGT-3’
ColX-Cre-Reverse-1:	5’-TACTGACCTGTCGTTTCC-3’
ColX-Cre-Reverse-2:	5’-GCATAACCAGTGAAACAGCATTGCTG-3’
ColX-Null-Forward:	5′-ACACAAAGGAGATATTGGCC-3′
ColX-Null-Reverse:	5′-AGGGGAGGAGTAGAAGGTGG-3′

### Generation of GFP/13del chimeric mice

For mouse superovulation, female mice of corresponding strains will be injected with follicle-stimulating hormone (5 IU/30 g body weight). 48 h later, the mice will be injected with hCG (5 IU/30 g body weight) to induce ovulation and will mate with the studs. For chimera mouse generation, an aggregation culture plate was prepared one day before the experiment, with many depressions and KSOM medium on the bottom of the plate. Superovulated M/M female mice were set to mate with homozygous ICR male mice ubiquitously expressing GFP (generated by crossing the CRE reporter mouse, Z/EG, and β-actin Cre transgenic mice^74^), and plugged female mice were sacrificed to collect dpc2.5 embryos in M2 medium. Collected embryos were removed from the zona by using Acid Tyrode, followed by washing with M2 medium, and placed into depressions in an aggregation culture plate at a 1:1 ratio in an incubator at 37 °C overnight. Wild-type ICR surrogate mothers were prepared at the dpc2.5 stage, and all the successful aggregations were transferred to the uterus of the surrogate mothers. Chimera mice were born after around 18 days post-implantation.

### Other mouse lines

In the generation of the Col10a1-Cre mouse, Cre recombinase followed by the Frt-flanked neomycin resistance gene (PGKneo) was inserted in exon 2 of the Col10a1 gene, extending from -2 070 to +7 680 of the cDNA (genomic) sequence. The PGKneo selection cassette was then removed by crossing to b-action-Flp mice.^14^ The HIF-1α-Flox mouse was purchased from The Jackson Laboratory. It has loxP sites on either side of exon 2 of the HIF-1α gene. When these HIF-1α-Flox mice are bred to mice that express Cre recombinase, the resulting offspring will have exon 2 deleted in the Cre-expressing tissues.^30^ The Col10a1-Null mouse was previously reported.^33^ Heterozygous female and male Col10a1-Null mice were bred and mated to generate wild-type and homozygous littermates. The C10-Atf4 transgenic mouse was previously reported.^20^

### Radiography of the mouse skeleton

Mice were anesthetized before radiography using the Senograph 600 T Senix HF x-ray machine (General Electric) at the Department of Diagnostic Radiology, Queen Mary Hospital, Hong Kong, China. The posture of sedated mice was fixed with sticky tape, and an aluminum step wedge was used as a guide for densitometry. High-resolution Kodak Diagnostic film for mammography was exposed to the appropriate radiation energy (for most of the time at 24 kV and 25 mA) at a magnification of 1.8. The X-ray films were developed by a film-processing machine.

### Skeletal preparation

Mice were sacrificed and fixed in absolute ethanol for 4 days. They were then cleared in acetone for 3 days. After rinsing with water, the skeletons were stained in Alcian blue/Alizarin red staining solution for 10 days. After rinsing again in water, the specimens were cleared in a clearing solution containing 1% (w/v) KOH until the soft tissues had been removed and the skeletons became clearly visible. To store the specimens, they were immersed in 50% (v/v), 80% (v/v), and finally 100% glycerol in a consecutive order overnight for each solution.

### Preparation of riboprobe for hybridization

The plasmid DNA containing the probe (Table 3) was linearized with the appropriate restriction enzyme and purified by phenol/chloroform extraction. In vitro transcription reaction was performed by mixing 1 μg of linearized template, with a transcription buffer supplemented with 10 mmol/L DTT, 250 μmol/L of ATP, CTP and GTP, 50 U of RNasin, 100 μCi of [α35S]- UTP or [α33P]-UTP and the appropriate RNA polymerase. The reaction mixture was incubated at 37 °C for 1 h. 1 μL of RNase-free DNase was added to the mixture and incubated at 37 °C for 30 min. A column buffer was added to stop the reaction, and 10 μg of yeast-tRNA was added to the probe before it was purified by a Sephadex G50 spin column. 1 μL of the purified probe was added to 2.5 mL of scintillation cocktail, and the radioactivity was estimated using a scintillation counter. The rest was precipitated with 0.5 volume of 6 mol/L ammonium acetate pH 5.3 and 2 volumes of ice-cold absolute ethanol. The labeled probe was pelleted at 16 000 × *g* at 4 °C for 30 min, then washed with 80% (v/v) ethanol and air-dried.

**Table 3:** Plasmid probes used for In situ hybridization

Target gene	Plasmid name (probe synthesis)	Description
*Col2a1*	pNJ61((EcoRI/T3; HindIII/T7)	0.5 kb cDNA contains exon 1 to 5B of mouse Col2a1.^14^
*Col10a1*	pRK26(EcoRI/Sp6; HindIII/T7)	0.35 kb cDNA of mouse Col10a1 gene that convers part of exon 3 and 3’ UTR.^14^
*Col10a1-13del*	pWF38 (EcoRI/T3; HindIII/T7)	A 64 bp fragment that corresponds to the Col10a1-13del sequence at the 13del region.
*Bip*	Bip (XhoI/T7; SpeI/T3)	Corresponds to position 9-1991 in mouse Bip mRNA (accession number AJ002387)^14^
*Ppr*	R15B (BamHI/Sp6; XbaI/T7)	2 kb cDNA of rat parathyroid hormone/ parathyroid hormone erelatd peptide gene.^78^
*Ihh*	Hh-14.1 (XbaI/T7; HindIII/T3)	1.8 kb EcoR1 cDNA fragment of mouse Indian hedgehog gene.^79^
*Mmp13*	M collase (HindIII/T7)	A 731 bp AvrII-HindIII fragment.^80^

### In situ hybridization on tissue sections

In situ hybridization was performed to detect the specific transcript in the growth plate and bone as previously described.^75^ The probes used are listed in the key materials table. Paraffin sections were dewaxed in xylene and rehydrated. Sections were then treated with proteinase K (10 μg/mL) for 5 min, washed with PBS and fixed in 4% paraformaldehyde for 3 min, followed by a rinse in double-distilled water and then treated in acetic anhydride [0.25% (v/v) in 0.1 mol/L triethanolamine, pH 8.0] for 10 min with stirring. Sections were rinsed with PBS, dehydrated with ethanol and air-dried. The [α35S]-UTP-labeled riboprobes were diluted in hybridization mix and heated to 80 °C for 2 min and applied to sections. The slides were placed horizontally in an airtight jar and incubated at 55 °C overnight. After hybridization, the slides were washed with wash buffer 15 mmol/L DTT, 2x SSC in 50% (v/v) formamide at 50 °C and treated with RNase A (20 μg/mL) and RNase T1 (50 U/mL) in NTE buffer at 37 °C for 20 min to digest unhybridized RNA. The slides were washed in wash buffer, followed by 2x SSC and 0.1x SSC. They were then dehydrated through a series of 30%, 80% (v/v) ethanol containing 0.3 mol/L ammonium acetate (pH 5.2), 100% ethanol, and air-dried.

### Immunohistochemistry and immunofluorescence staining on sections

Bones were fixed in 4% (v/v) paraformaldehyde, and if necessary, demineralized in 0.5 mol/L EDTA (pH 8.0) containing 0.2% (v/v) formaldehyde before embedding in paraffin. Paraffin sections were deparaffinized and rehydrated through a decreasing series of alcohol concentrations. The endogenous peroxidase activity was quenched with 3% (v/v) H_2_O_2_ in methanol for 20 min, which was followed by blocking the non-specific protein adsorption with an appropriate blocking serum/reagent for 30 min. It was subsequently probed with an appropriately diluted primary antibody overnight at 4 ^o^C. The next step was incubation with a horseradish peroxidase-conjugated secondary antibody (which recognized the primary antibody) for 30 min. Immunohistochemistry and immunofluorescence staining were performed using antibodies specific for Collagen X and 13del mutant Collagen X (Generated by our lab^14^), Cyclin D1 (Santa Cruz, Cat#sc-753), Xbp1s (BioLegend, Cat# 647502), CD31 (Abcam, Cat# ab28364), HIF1a (SANTA CRUZ BIOTECH, Cat# sc-10790), HIF2a (SANTA CRUZ BIOTECH, Cat# sc-28706), p53 (E-19) (SANTA CRUZ BIOTECH, Cat# sc-1315), Tap63 (NOVUS BIOLOGICALS, Cat# NBP3-11703), ΔNp63 (BioLegend, Cat# 619001), EDEM (Provided by Kumamoto University, Japan), iASPP (Sigma, Cat# A4605), ASPP2 (Provided by Dr. Xin Lu from University of Oxford), ATF4 (SANTA CRUZ BIOTECH, Cat# sc-200), p57Kip2 (SANTA CRUZ BIOTECH, Cat# sc-56341), EF5-Cy3 regular stain antibody and EF5-competed stain antibody (Provided by Dr. Cameron Koch from University of Pennsylvania Department of Radiation Oncology), Map-LC3B (MBL life science, Cat# PM036), CHOP (SANTA CRUZ BIOTECH, Cat# sc-166682), GADD34 (SANTA CRUZ BIOTECH, Cat# sc-794), ATF3 (SANTA CRUZ BIOTECH, Cat# sc-81189), 488 Goat Anti-Rabbit IgG (Life technologies, Cat# A-11008) were used. For immunofluorescence staining, the H_2_O_2_ quenching step was not necessary, and secondary antibodies conjugated with a fluorophore were used instead of the HRP-conjugated ones.

### Toluidine blue staining

Sections were dewaxed in xylene and rehydrated through a descending ethanol series and stained with 1% (w/v) toluidine blue O (Sigma) in 50% (v/v) isopropanol at 37 °C for 30 min. Sections were destained in absolute isopropanol, dehydrated in xylene, and mounted.

### Hematoxylin and eosin (H&E) staining

Paraffin sections were placed in a 60 °C oven for half an hour to remove excess paraffin, then dewaxed in xylene (2×5 min) and rehydrated through descending ethanol concentration, from absolute to 30% (v/v) ethanol and then in distilled water. Sections were first stained in Harris’s hematoxylin for 5 min, rinsed twice in distilled water until the water was clear, and then differentiated in acid alcohol [1% (v/v) HCl in 70% ethanol (v/v)] for 10 sec, then developed a blue color in freshly prepared 2% (w/v) NaHCO_3_ for 2 min. They were washed twice in distilled water and stained in Eosin solution for 4 min. Sections were dipped into tap water before they were placed in 95% (v/v) EtOH for 3 min, and then in 100% EtOH (2×5 min), to be dehydrated and cleared in xylene. Stained slides were mounted in DEPEX mounting medium (BDH) with coverslips.

### 5-Bromo-2’-deoxy-uridine (BrdU) Labeling and Detection

Mice were injected intraperitoneally with 200 µg of BrdU per g of body weight 2 h before sacrifice. Incorporated BrdU in cell nuclei was detected using a BrdU staining kit (Zymed Laboratories) according to the manufacturer’s protocol.

### Terminal deoxynucleotidyl transferase dUTP-mediated Nick End Labeling (TUNEL) assay

Apoptotic cells were detected using the In Situ cell death detection Kit, Fluorescein (Roche) according to the manufacturer’s instructions. The rehydrated sections were treated with proteinase K (2-10 μg/mL) for 15 min at RT and incubated with a reaction mix containing terminal deoxynucleotidyl transferase (TdT) and digoxigenin-labeled nucleotides. The sections were then counterstained with propidium iodide (PI) to display the nuclei.

### EF5 distribution analysis

To study EF-5 distribution, 10-day-old mice or pregnant females (for prenatal stage mice) were intraperitoneally injected with 10 mmol/L EF5 at 1% body weight. Hind limbs were dissected after 4 h of injection. Frozen sections were prepared for staining. Slides were air-dried for at least 30 min and then fixed in ice-cold acetone for 10 min, followed by air-drying again for 30 min. Sections were incubated in mouse IgG (diluted in PBS) for blocking overnight at 4 °C. Blocking solution was removed, and slides were dipped in ttPBS (4 g Tween 20; 0.04 g Thimerosal; 0.1 g Sodium azide diluted in 50 mL 20 X PBS) and moisture blotted away. Sections were incubated in Cy3-conjugated ELK-351 antibody (control sections were incubated in Cy3-conjugated ELK-351 antibody with EF5 drug) for 5 h at room temperature. The staining solution was removed, and sections were washed with ttPBS (3×45 min). Control sections were washed with ttPBS with EF5 (4×2 min and 1×45 min), followed by 45 min washing in PBS for the last time. Images were taken immediately after the staining.

### Administration of Chloroquine diphosphate

Chloroquine diphosphate (dissolved in PBS) was administered to mice at a dosage of 60 mg/kg body weight by intraperitoneal injection. Mice assigned to the control group were treated with PBS. Mice were sacrificed 4 h later, and bones were harvested and fixed in 4% (v/v) PFA for analysis. For quantification of LC3 puncta-positive cells, the hypertrophic zone was divided into three zones: upper, middle, and lower regions according to cell morphology and anatomical position. Hypertrophic chondrocytes containing two or more clearly distinguishable LC3-positive puncta were defined as LC3 puncta-positive cells. For a defined region of each zone, the percentage of LC3 puncta-positive hypertrophic chondrocytes was calculated as the percentage of LC3 puncta-positive hypertrophic chondrocytes over the total number of hypertrophic chondrocytes. The same threshold and scoring criteria were applied to all control and chloroquine-treated samples.

### Measurement of coxa vara

Mice were anesthetized to facilitate radiography, and all were positioned such that the greater trochanters of both femurs were clearly seen on the radiographs. On the radiographs, axes for the femur neck and shaft were drawn, and the obtuse angles between these two axes were measured as previously described.^14,15^

### Sciatic neurectomy

Surgery was performed after anesthetizing the mice with a subcutaneous injection of one part fentanyl-fluanisone (Hypnorm®, Janssen Inc., USA), one part midazolam (Dormicom®, Roche Ltd., Switzerland), and 5.5 parts autoclaved distilled water. Unloading of the limbs in 3-week-old mice (*n* = 3 for each genotype) was performed by sciatic neurectomy as described.^76^ 0.5 cm of the sciatic nerve on the femur region of the left hindlimbs was removed, whereas that on the right hindlimbs was left unoperated as a control. The wounds were immediately closed with sterile sutures, cleaned with povidone-iodine disinfectant and 70% (v/v) isopropyl alcohol, and the operated mice were administered a painkiller in drinking water (0.1 mL flunixin in 200 mL tap water). After sciatic neurectomy, mice are unable to stand, walk, or jump with their operated limbs. However, they can still have access to food and water with the remaining three limbs. Radiographs were taken one week after the operation, and the *coxa vara* condition was assessed. Mice were sacrificed, and bones were collected and fixed for histological analyses.

### Image processing and analysis

EF5 signal distribution was analyzed using ImageJ. Composite images presented in Fig. 2e, Fig. 3a, c, and d, Fig. 4c and d, Fig. 5b, Fig. 6d and e, Fig. S8, and Fig. S11 have been generated by merging individual images obtained from independent channels using Photoshop CS5 software.

### Statistical analysis

Statistical significance was calculated with either Student’s t-test provided by Microsoft Excel or an ordinary one-way ANOVA analysis from GraphPad Prism 9. (*P value < 0.05; **P value < 0.01; ***P value < 0.001)

## Supplementary information

Supplemental Figure S1–S14

Supplemental Figure legends
