# Supplementary material for: Integrated stress signaling pathways promote adaptive reprogramming for chondrocyte survival in Metaphyseal Chondrodysplasia Type Schmid
Source: Bone Res. 2026 Oct 16;14:106. doi: 10.1038/s41413-026-00587-5 (PMC13675185; doi:10.1038/s41413-026-00587-5)

Supplemental Fig. S1 Skeletal preparation of newborn mice

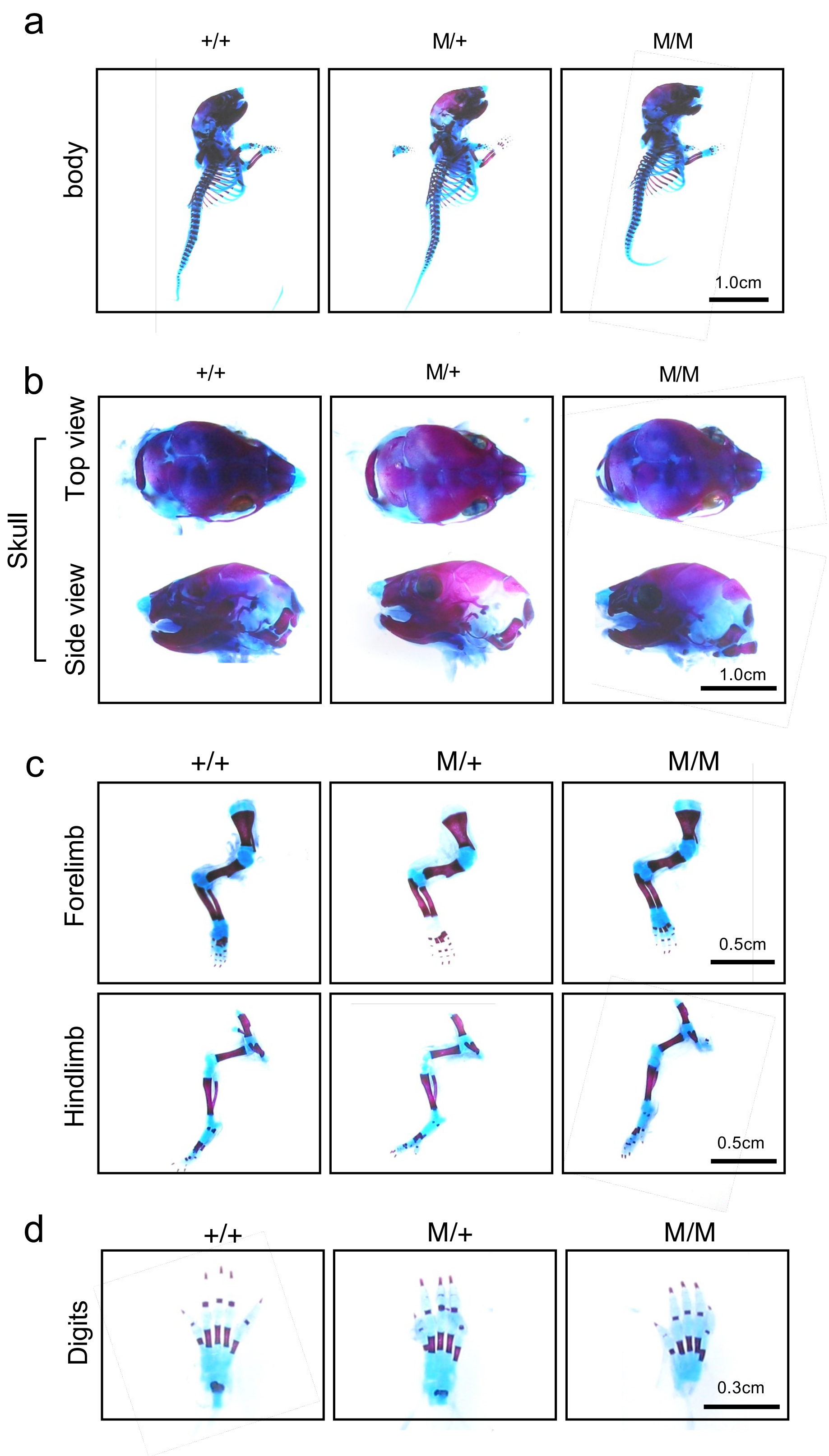

Supplemental Fig.S2 X-ray of vertebral bodies

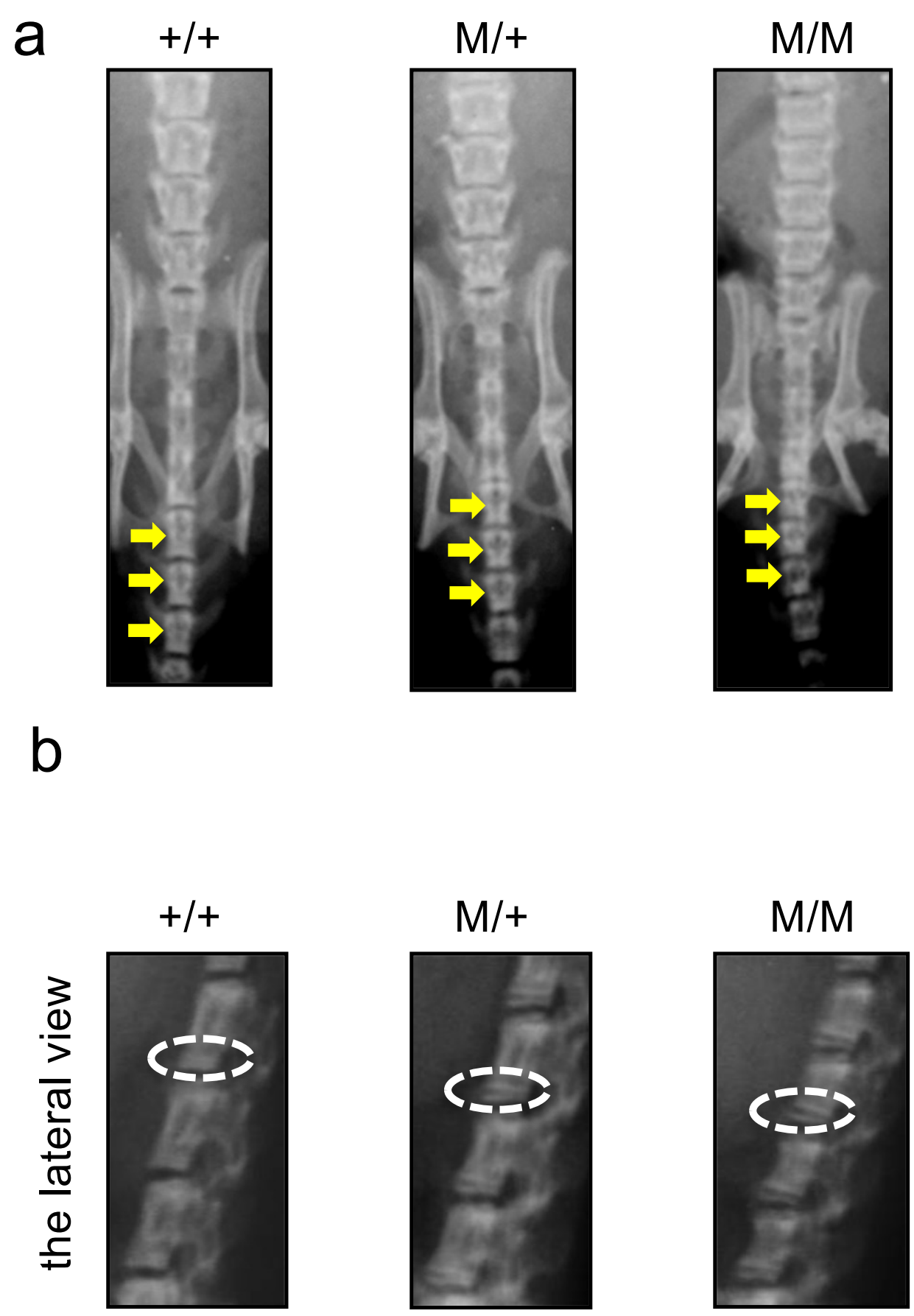

Supplemental Fig. S3 Shortening of digits in 13del-MCDS mice

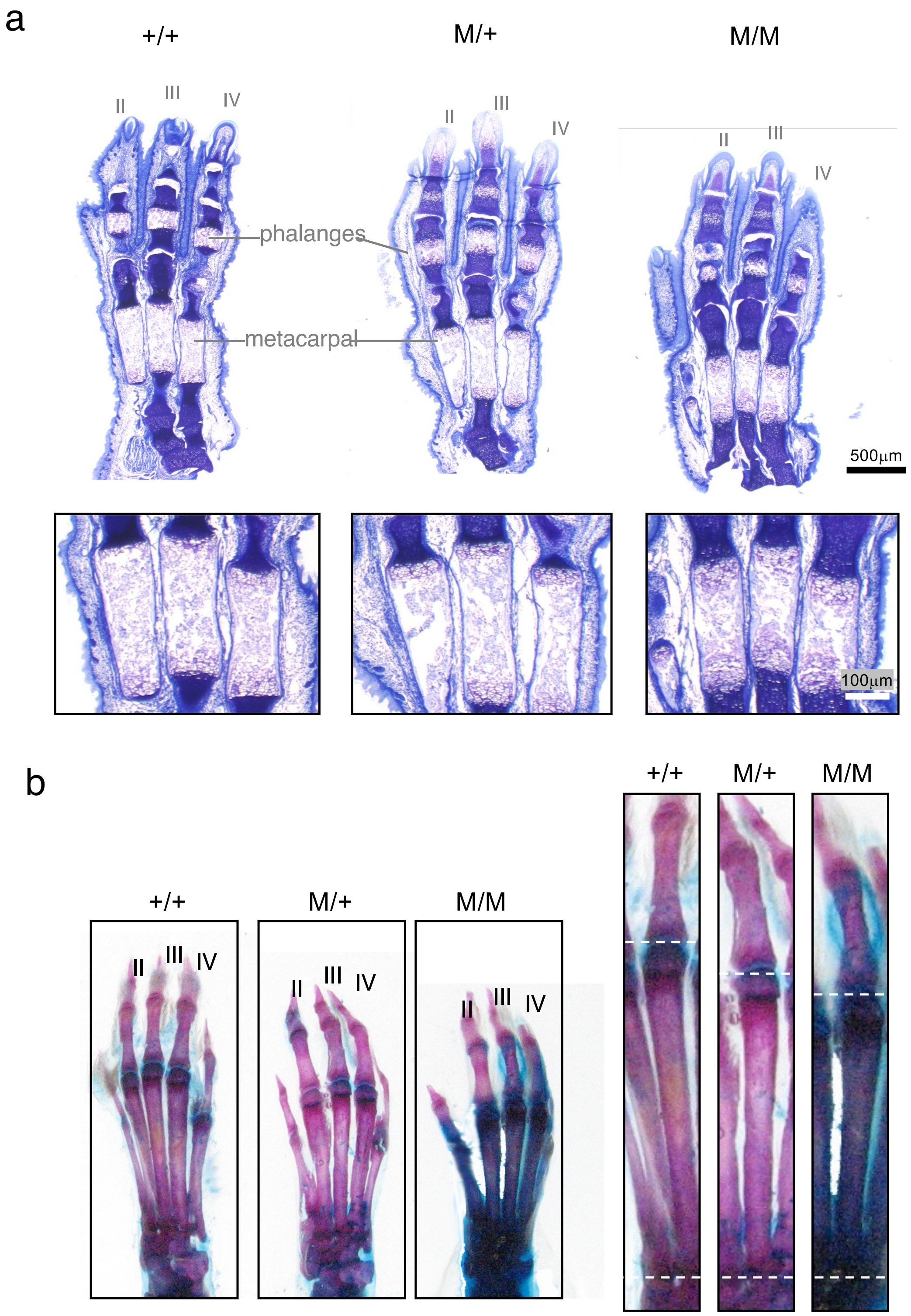

Supplemental Fig. S4 Abnormalities in the rib cages in 13del-MCDS mice

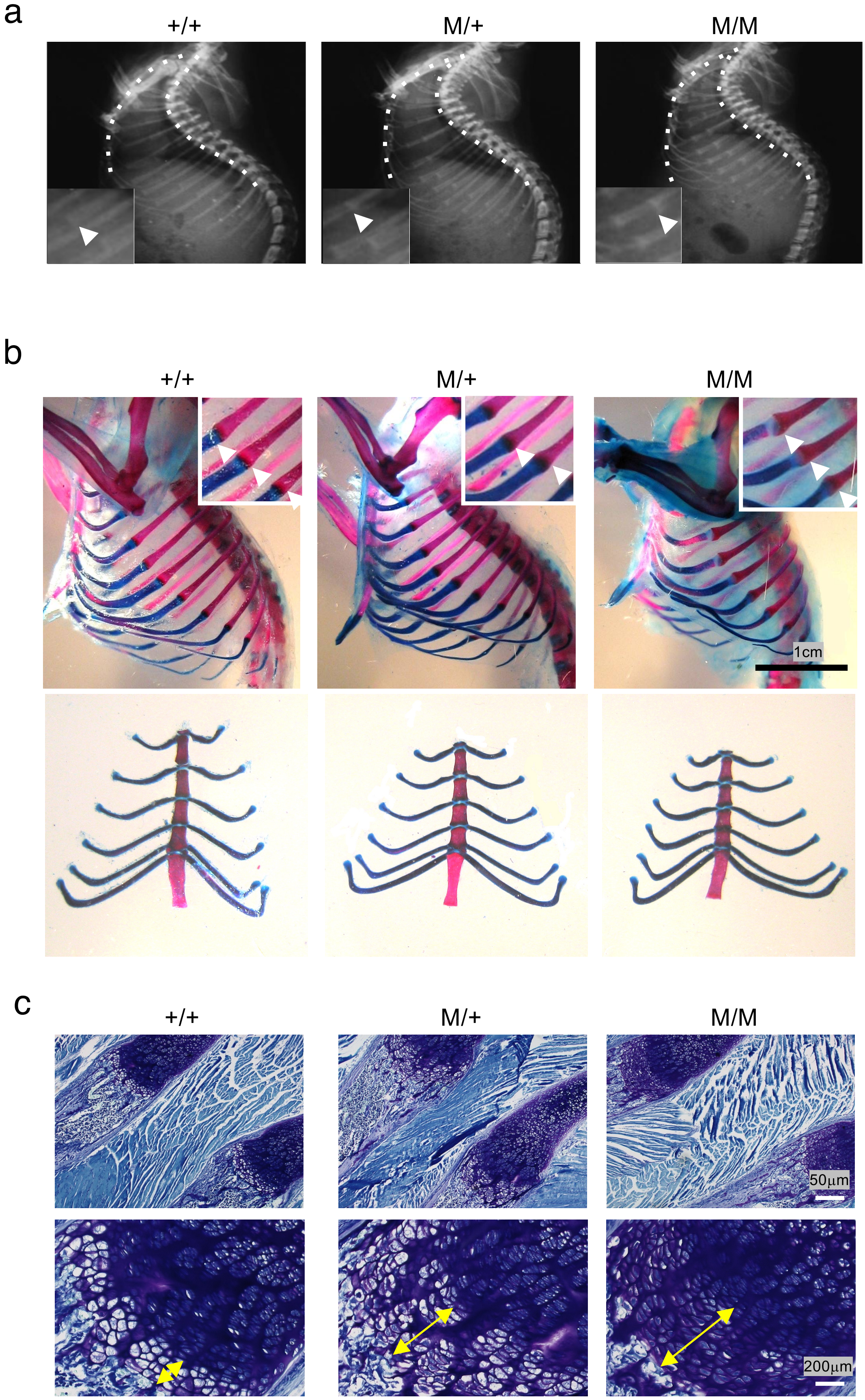

Supplemental Fig. S5 Abnormal femoral head in 13del-MCDS mice

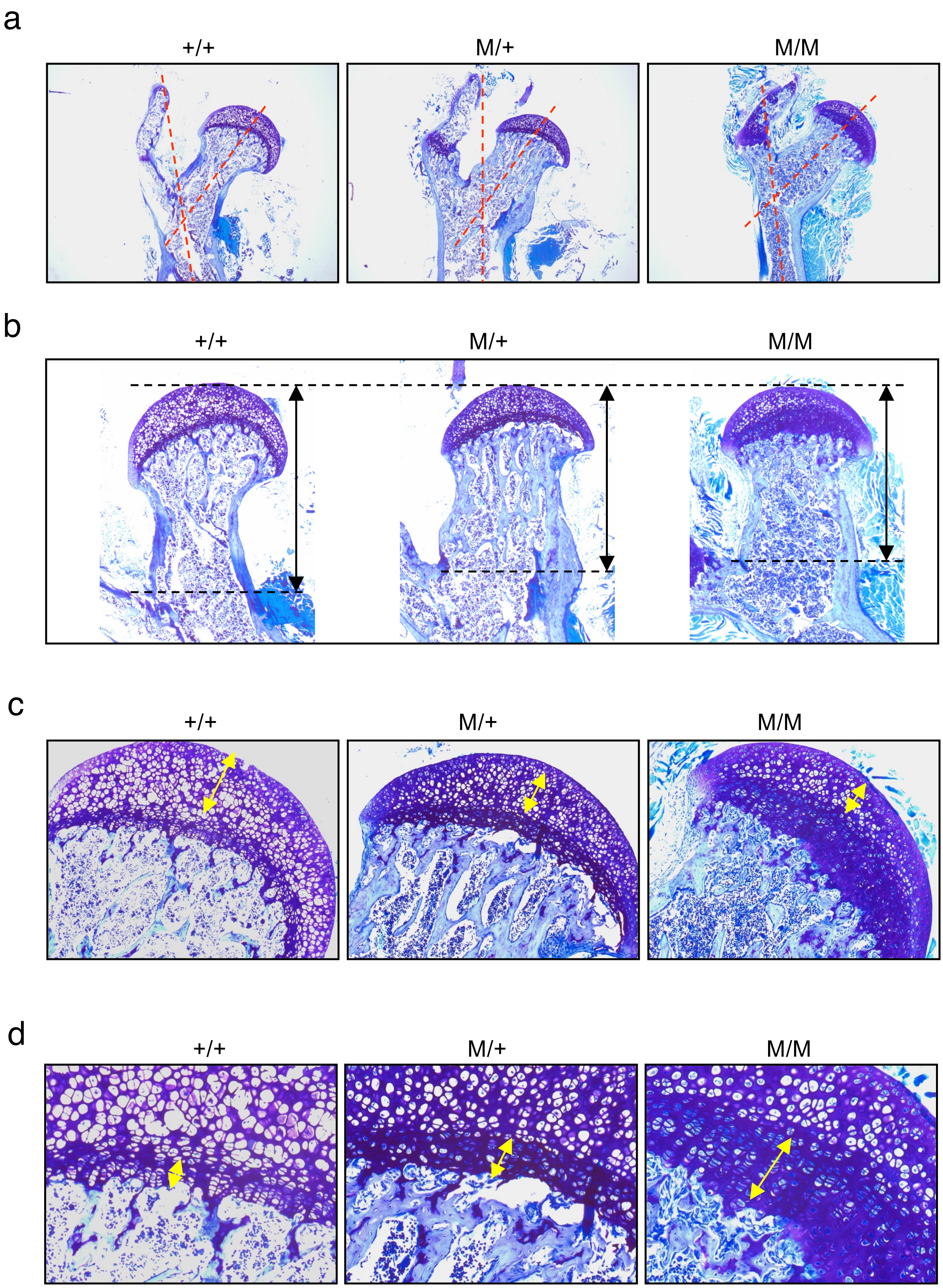

Supplemental Fig. S6. Unloading of hindlimbs leads to reduced growth plate expansion in 13del-MCDS mice

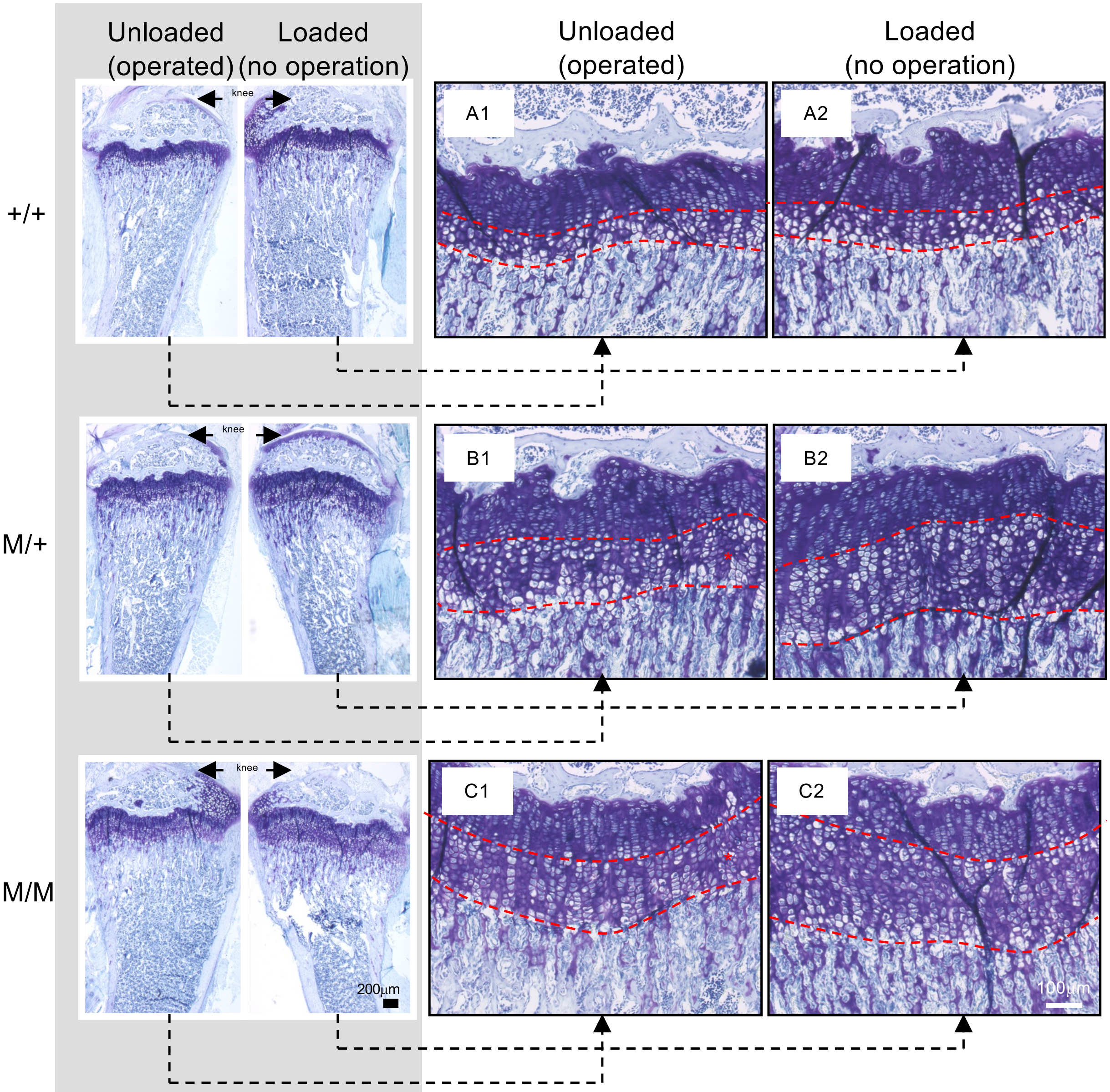

Supplemental Fig. S7 UPR activation in the hypertrophic chondrocytes of 13del-MCDS mice

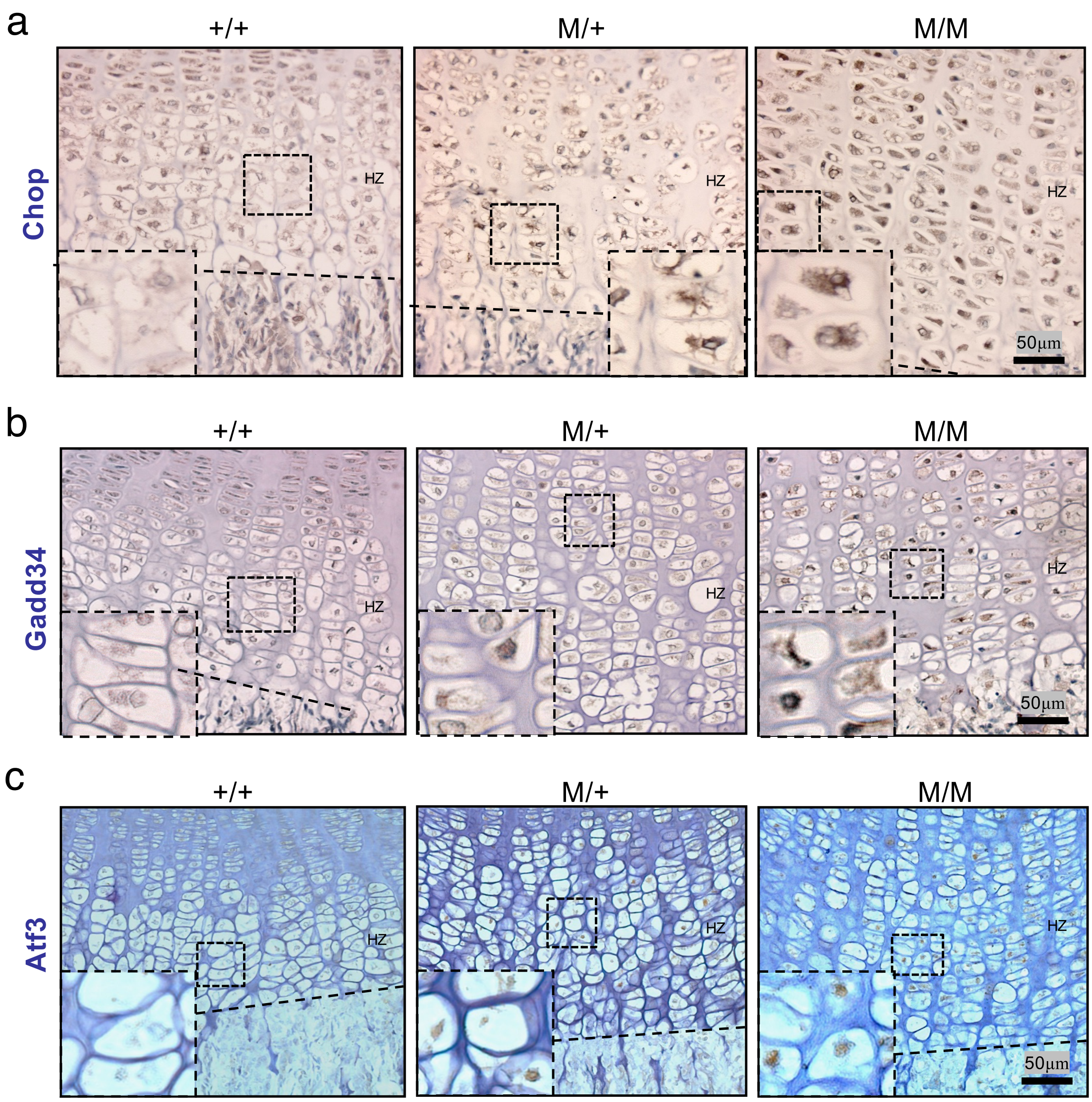

Supplemental Fig. S8 TUNEL- positive cells were not increased in the 13del-MCDS hypertrophic zone at P10

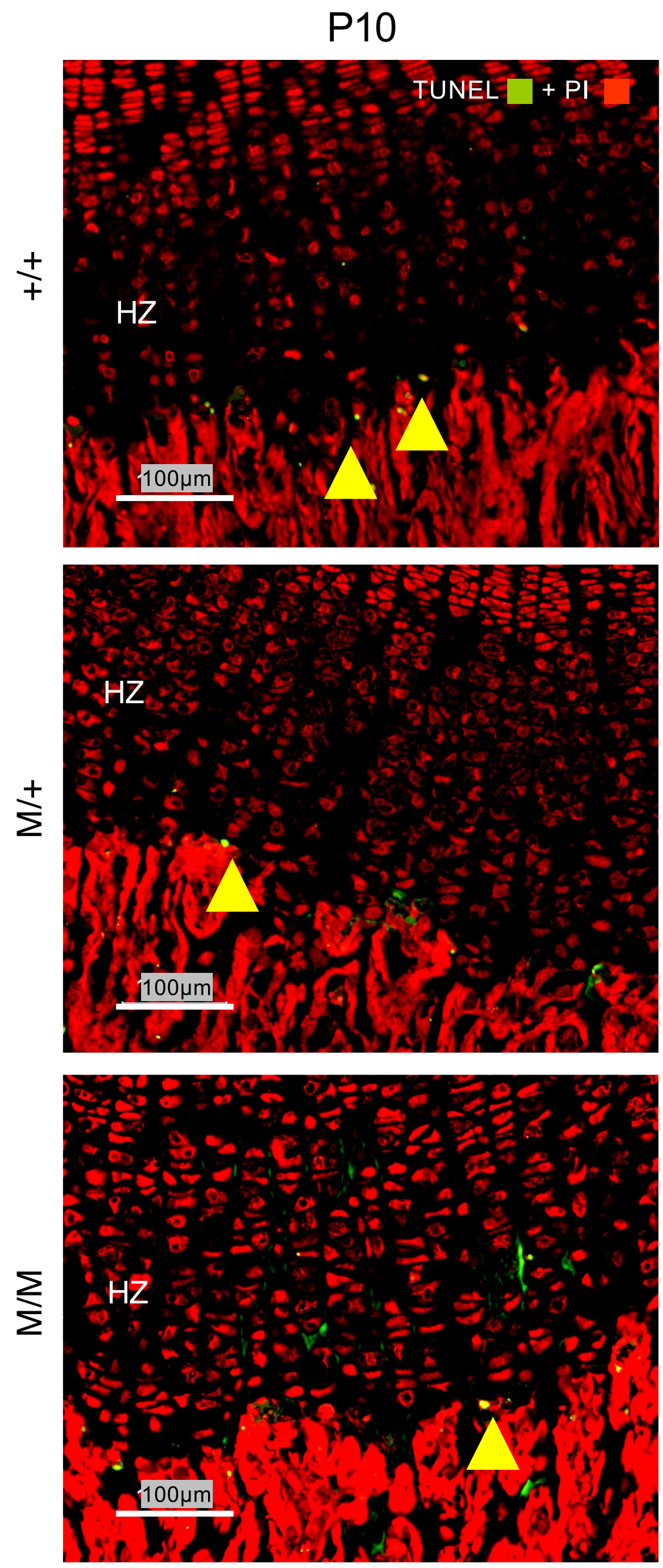

Supplemental Fig. S9 ***In situ hybridization*** **of Ppr and Mmp13 mRNA in growth plate of 13del-MCDS mice at P0**

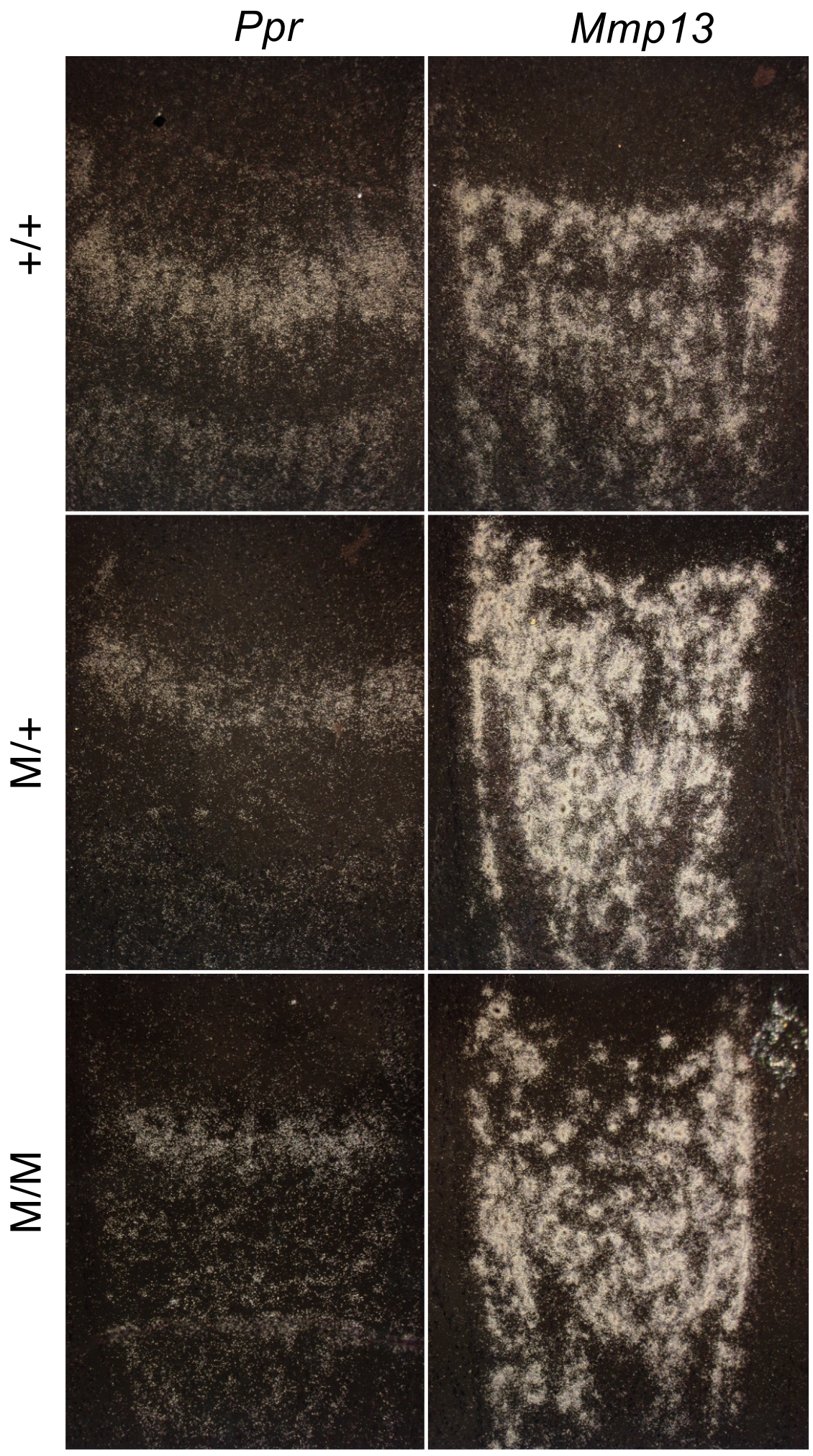

Supplemental Fig. S10 P57^{Kip2} Immunohistochemistry staining in the growth plate of 13del-MCDS mice

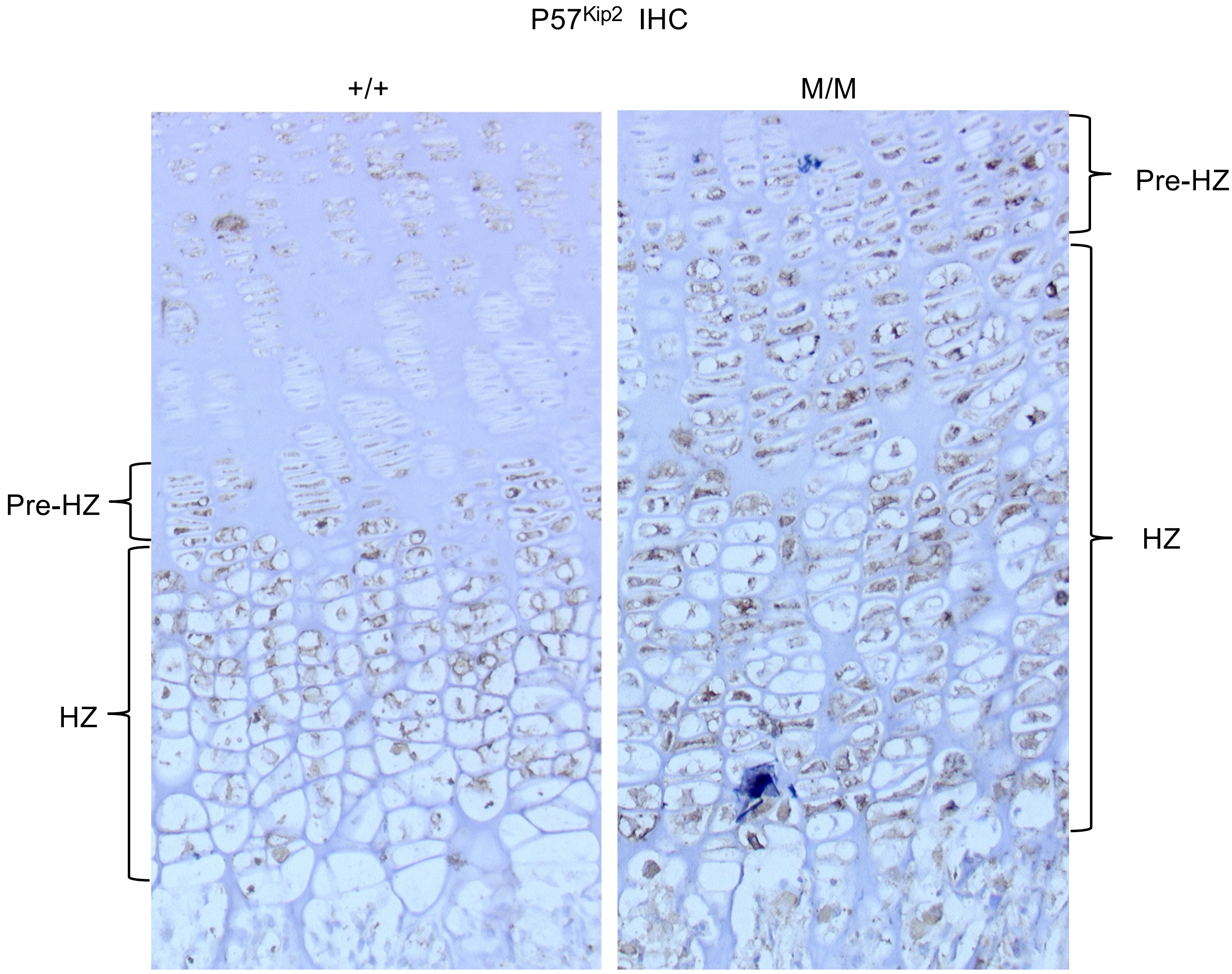

Supplemental Fig. S11 Increased hypoxic hypertrophic chondrocytes in the 13del-MCDS distal growth plate of the tibia at P10

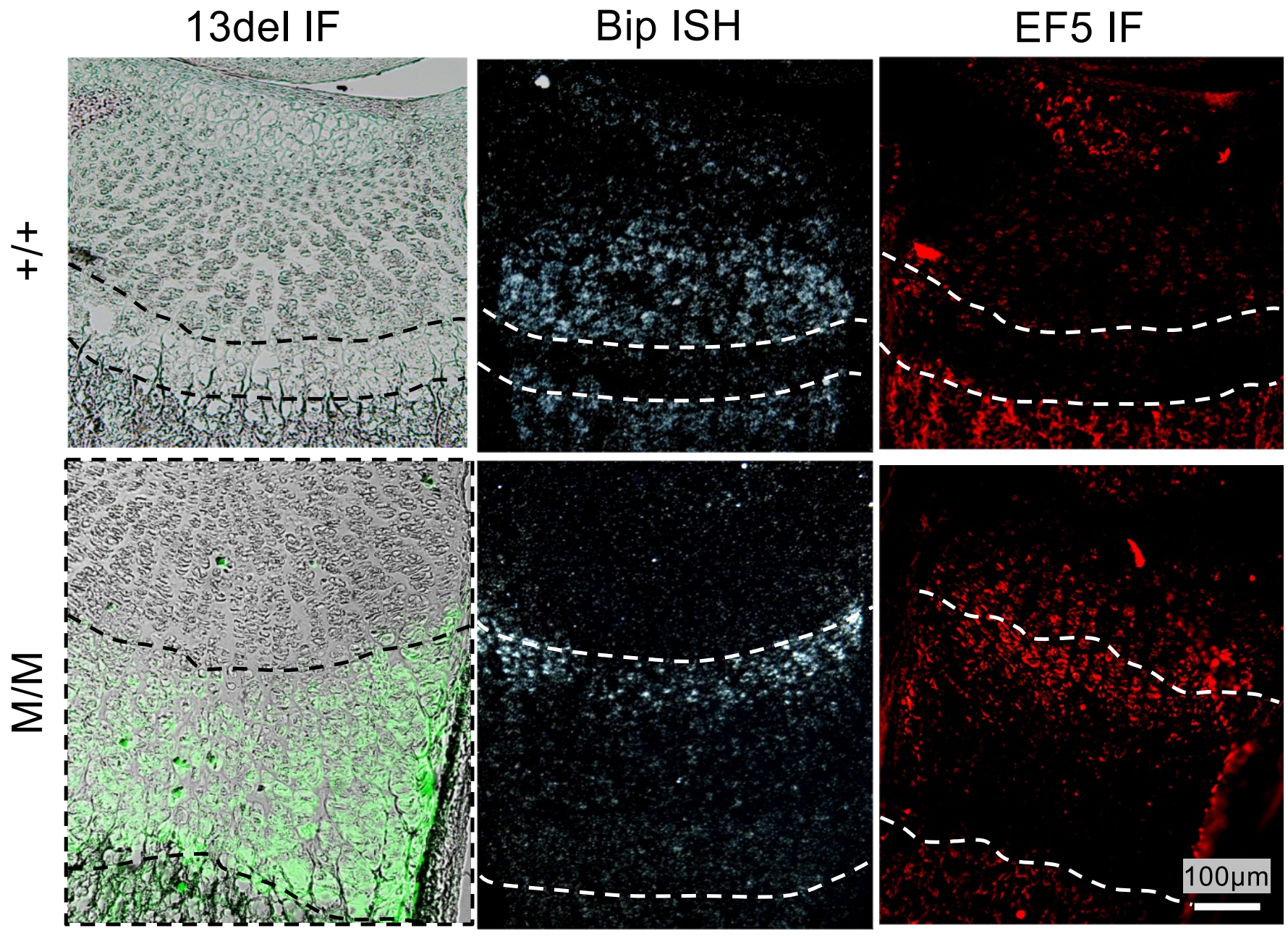

Supplemental Fig. S12 Conditional knock out of Hif1a in the hypertrophic chondrocytes of 13del-MCDS mice

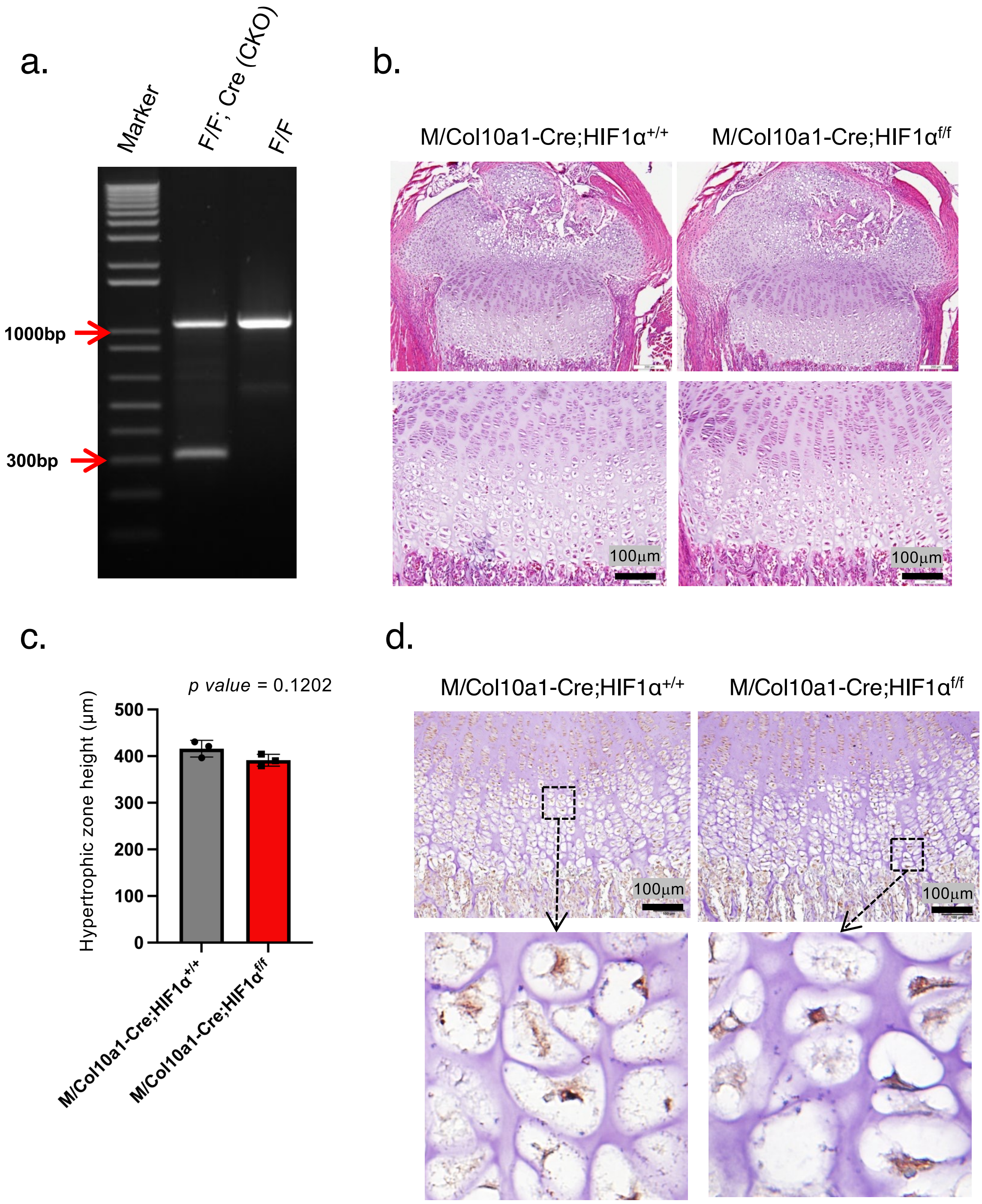

Supplemental Fig. S13 Detection of hypoxia in the growth plate of Col10a1-Null-HO mice.

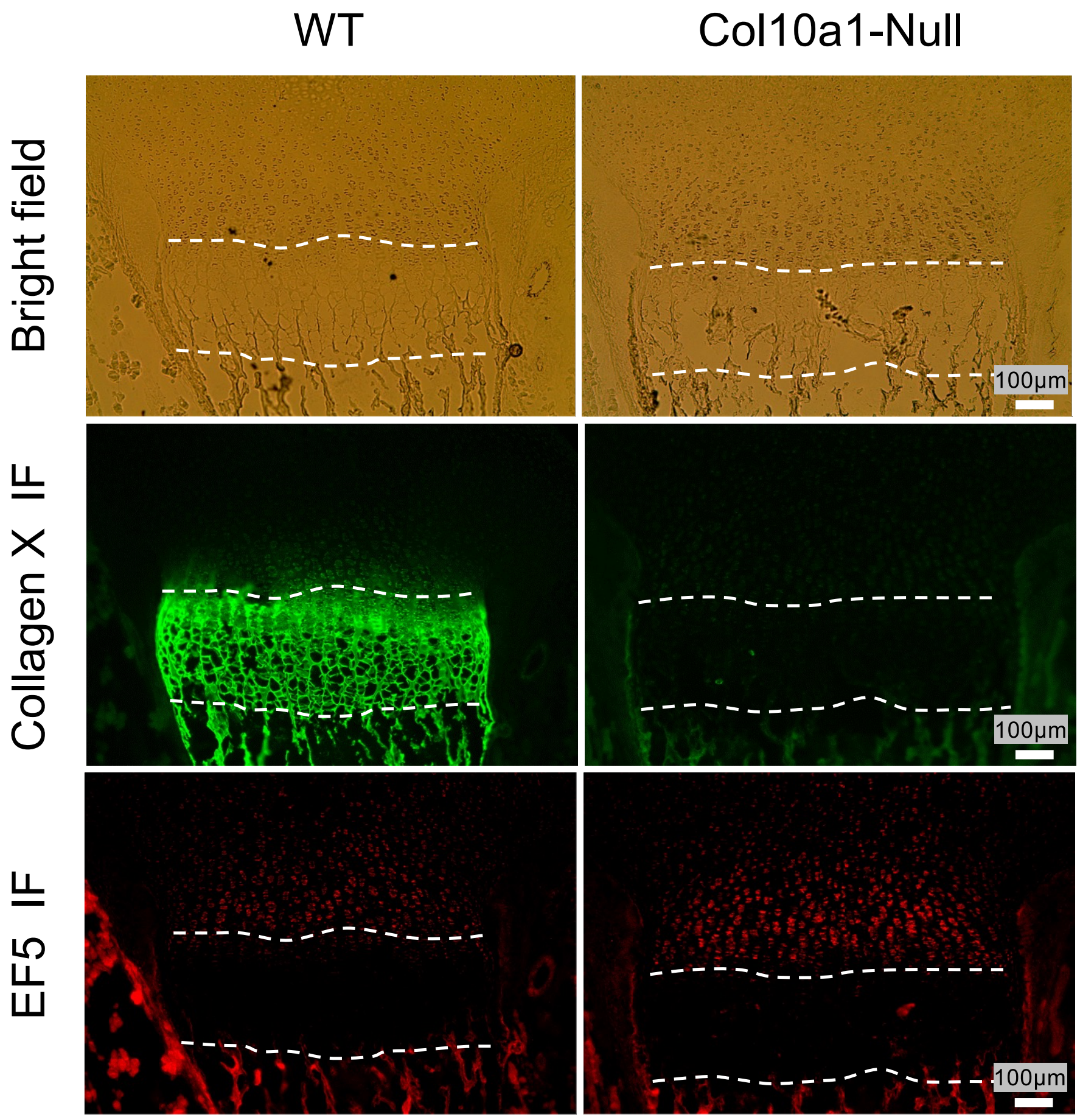

Supplemental Fig. S14 Hypoxia and HIFs pathways are activated in talus hypertrophic chondrocytes of MCDS mice.

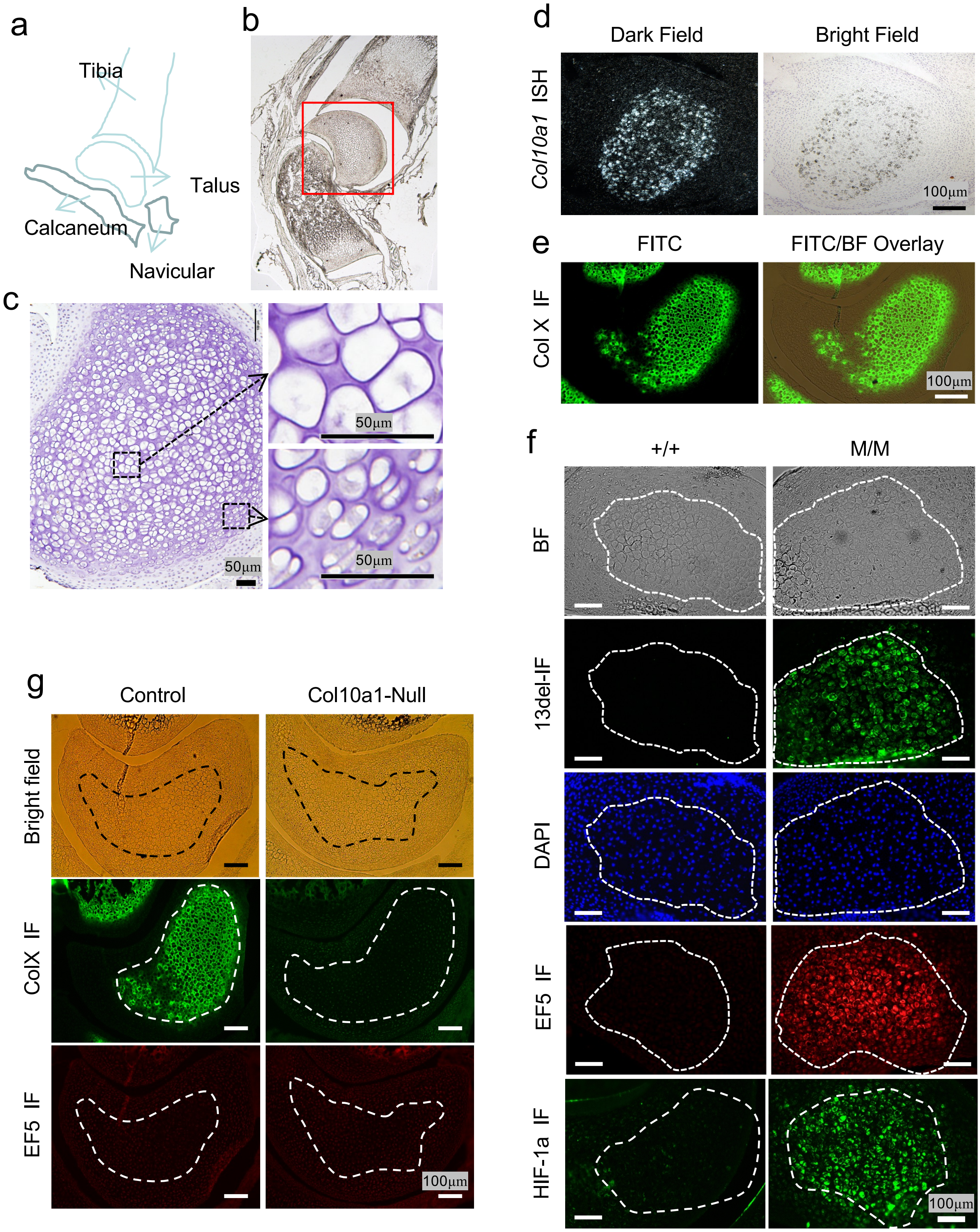

Supplement: Supplementary file 1 [file 41413_2026_587_MOESM1_ESM.pdf]
