# Supplementary material for: Integrated stress signaling pathways promote adaptive reprogramming for chondrocyte survival in Metaphyseal Chondrodysplasia Type Schmid
Source: Bone Res. 2026 Oct 16;14:106. doi: 10.1038/s41413-026-00587-5 (PMC13675185; doi:10.1038/s41413-026-00587-5)
Supplement: Supplementary file 2 [file 41413_2026_587_MOESM2_ESM.docx]

**Supplemental Figure S1. Skeletal preparation of newborn mice**

Alcian Blue and Alizarin Red staining for skeletal preparation of littermates of newborn 13del-MCDS mice. (a) The body size of M/M and M/+ mice was comparable to that of +/+ littermates. (b) Top view and side view of the skull. (c) The skeletal forelimbs and hindlimbs of M/M and M/+ mice compared to +/+ littermates. (d) The skeletal feet of M/M and M/+ mice compared to +/+ littermates.

**Supplemental Figure S2. X-ray of vertebral bodies**

Radiographs of the ventral and lateral views of the spine of 8-week-old mice. (a) The vertebral bodies (arrows) in the spine of the mutant mice were shortened. (b) From the lateral view of the spine, subtle flaring of metaphyses (dotted circles) in the vertebral bodies was observed.

**Supplemental Figure S3. Shortening of digits in 13del-MCDS mice**

(a) Paraffin sections of feet from 10-day-old mice were stained with toluidine blue. The digits in M/+ mice were comparable to those in +/+ mice. The tubular bones in the feet, phalanges, and metacarpals were shortened in M/M mice. The distance between the growth plates in the same metacarpal bones was reduced. (b) Skeletal preparation of feet from 4-week-old mice. The metacarpals were shortened in 4-week-old M/+ and M/M mice compared to their +/+ littermates.

**Supplemental Figure S4. Abnormalities in the rib cages in 13del-MCDS mice**

(a) Radiographs of the thorax (rib cage) of 4-week-old mice. M/M mice displayed slight narrowing of the rib cage. (b) Skeletal preparation of the rib cages from 4-week-old mice. The rib cages in the M/M mice were narrowed. Note that the chondro-osseous junction of the rib bones was bulged in M/+ and M/M mice (white arrows). Scale bar= 1cm. (c) Paraffin sections of ribs from 4-week-old mice were stained with toluidine blue. Scale bar= 50μm. Expansion of the hypertrophic zone (yellow arrows) in the growth plates of the ribs in M/M mice was observed. Scale bar= 200μm.

**Supplemental Figure S5. Abnormal femoral head in 13del-MCDS mice**

Histological analysis by toluidine blue staining in paraffin sections from 4-week-old M/M mice and their littermate controls. (a) The femoral heads of M/M mice were malformed. (b) The dome shape of the femoral head of M/+ and M/M mice is flattened compared to the round-shaped femoral head of +/+ mice. The femoral heads were shortened in both M/+ and M/M mice (double-sided arrows). (c) The articular cartilage was thinner in M/+ and M/M mice compared to that in +/+ mice. (d) The growth cartilage under the articular cartilage of the femoral head in M/+ and M/M mice is thicker compared to that in +/+ mice.

**Supplemental Figure S6.** **Unloading of hindlimbs leads to reduced growth plate expansion in 13del-MCDS mice.**

Histological analysis by toluidine blue staining in paraffin sections from hindlimbs of 4-week-old mice (sacrificed one week after sciatic neurectomy) in the sciatic neurectomy experiments. In +/+ mice, the hypertrophic zone (red dotted lines bounded) in the unloaded limb (A1) was comparable to that of the control limb (A2). In mutant mice, the hypertrophic zone in the unloaded limbs was less expanded (*, M/+: B1 & M/M: C1) than the loaded control limbs (M/+: B2 & M/M: C2).

**Supplemental Figure S7.** **UPR activation in the hypertrophic chondrocytes of 13del-MCDS mice**

Immunohistochemistry staining on the proximal tibia growth plate in paraffin sections from 10-day-old mice. (a) Chop. (b) Gadd34. (c) Atf3. Insets show higher-magnification images of HCs. HZ=hypertrophic zone. Scale bar = 50μm.

**Supplemental Figure S8.** **TUNEL- positive cells were not increased in the 13del-MCDS hypertrophic zone at P10**

TUNEL assay (green) was performed on 10-day-old tibial paraffin sections counterstained with propidium iodide (red). Apoptotic cells (yellow arrows pointed) were present at the chondro-osseous junction in +/+, M/+, and M/M mice. Scale bar = 100μm

**Supplemental Figure S9. *In situ hybridization* of Ppr and Mmp13 mRNA in the growth plate of 13del-MCDS mice at P0**

*In-situ* hybridization (ISH) of Ppr and Mmp13 mRNAs on paraffin sections of proximal tibial growth plate from newborn M/M mice and their littermate controls.

**Supplemental Figure S10. P57^Kip2^ immunohistochemistry staining in the growth plate of 13del-MCDS mice**

Immunohistochemistry (IHC) staining on the proximal tibia growth plate in paraffin sections from 10-day-old M/M mice and their littermate controls. Pre-HZ: pre-hypertrophic zone; HZ: hypertrophic zone. Scale bar=100μm.

**Supplemental Figure S11. Increased hypoxic hypertrophic chondrocytes in the 13del-MCDS distal growth plate of the tibia at P10**

13del antibody (Dilution, 1:400) immunofluorescence (IF) staining (left column) and EF5 staining (right column) were applied on tibia frozen sections of 10-day-old M/M mice and +/+ littermates injected with EF5. *In situ* hybridization (ISH) (middle column) was performed on paraffin sections of 10-day-old M/M mice and +/+ littermates, using probes detecting BiP mRNA. The dotted line indicated the hypertrophic zone. Scalebar=100μm.

**Supplemental Figure S12. Conditional knock out of Hif1a in the hypertrophic chondrocytes of 13del-MCDS mice**

(a) Recombination of HIF1a floxed alleles in the genomic DNA of Col10Cre target tissues (hypertrophic zones of the growth plates). PCR analysis of the genomic DNA from hypertrophic zone dissected from the tibia of M/Col10a1Cre; HiF1α^F/F^ mice showed that the recombined bands (~300bp) were detectable in the hypertrophic zone of Hif1a conditional knock-out mice, but not in their control littermates. (b) H&E staining of paraffin sections of proximal tibia growth plate from 10-day-old Hif1a conditional knock-out 13del-MCDS mice and their controls. Scale bar=100μm (c) Measurements of the height of the hypertrophic zone of the tibia proximal growth plate. N=3 mice. (d) Immunohistochemistry staining of HIF1a protein on the proximal tibia growth plate in paraffin sections from 10-day-old mice. High levels of HIF1α protein can still be detected compared with M/Col10a1Cre; Hif1α^F/F^ mice, making this assessment inconclusive. Scale bar=100μm

**Supplemental Figure S13. Detection of hypoxia in the growth plate of Col10a1-Null-HO mice.**

EF5 assay was applied to 10-day-old Col10a1-null-HO mice and their wild-type littermates. EF5 staining and ColX immunofluorescence staining (antibody dilution: 1:1000) were applied to tibia growth plate cryosections. Scale bar=100μm. The dotted lines indicate the hypertrophic zone.

**Supplemental Figure S14. Hypoxia and HIFs pathways are activated in talus hypertrophic chondrocytes of 13del-MCDS mice.**

(a) Mid-longitudinal internal view through the tibia demonstrating the anatomy of the talus. (b) Cryo-sections contained talus cartilage for staining. Red box indicates the talus cartilage being observed. (c) Hematoxylin staining on paraffin talus cartilage section. Dotted boxes indicate the regions of higher magnification shown on the right side. Scale bar=50μm. (d) In situ hybridization of Col10a1 mRNA was performed on talus cartilage paraffin sections of 10-day-old wild-type mice. Scale bar=100μm. (e) ColX immunofluorescence staining (antibody dilution: 1:1000) was performed on talus cartilage cryo-sections of 10-day-old wild-type mice. Scale bar=100μm. (f) 13del-specific antibody (antibody dilution: 1:400) and HIF1a (antibody dilution: 1:100) immunofluorescence staining and EF5-Cy3 staining were performed on talus cartilage cryo-sections of 10-day-old M/M mice and their littermate controls. The dotted lines indicate the region of HCs. Scale bar=100μm. (g) Detection of hypoxia in talus cartilage of Col10a1-Null-HO mice. EF5 staining and ColX immunofluorescence staining (antibody dilution:1:1000) were applied to talus cartilage cryo-sections from 10-day-old Col10a1-Null-NO mice and their littermate controls. The dotted lines indicate the region of HCs. Scale bar=100μm.
